# Uncovering the species diversity of subterranean rodents at the end of the World: three new species of Patagonian tuco-tucos (Rodentia, Hystricomorpha, *Ctenomys*)

**DOI:** 10.7717/peerj.9259

**Published:** 2020-05-29

**Authors:** Pablo Teta, Guillermo D’Elía

**Affiliations:** 1 División Mastozoología, Museo Argentino de Ciencias Naturales “Bernardino Rivadavia”, Buenos Aires, Argentina; 2 Instituto de Ciencias Ambientales y Evolutivas, Universidad Austral de Chile, Valdivia, Chile

**Keywords:** Caviomorpha, Octodontoidea, Taxonomy, Patagonia

## Abstract

*Ctenomys* Blainville 1826 is one of the most diverse genera of South American caviomorph rodents. Currently, six species of this genus are reported from Patagonia, south of 42°S. In this contribution, we assessed the taxonomic status of several populations from eastern and central Chubut province, northern Patagonia. Based on phylogenetic analyses of DNA sequences, morphology assessment (qualitative and quantitative), and previously published karyological data, we describe three new species of this genus, one formed by two subspecies, endemic to northern Patagonia. In addition, we include *C. coyhaiquensis* Kelt and Gallardo 1994 into the synonymy of *C. sericeus* J.A. Allen 1903. Finally, we discussed the need for additional integrative approaches, including field collection of specimens, to better understand the diversity of this highly speciose rodent genus.

## Introduction

The fact that species play a central role in all areas of comparative biology is well recognized (e.g.,  Coyne & Orr, 2004). As such, inaccurate species delimitation schemes mislead our understanding and characterization of nature (Sites Jr & Marshall, 2003) at the time that potentially hamper some strategies aimed to preserve biodiversity (Thomson et al., 2018). Given the pivotal role that species play in biology, several competing species concepts have been proposed, each centering on distinct putative species defining properties (Sites Jr & Marshall, 2004). A conceptual advance was the proposition of the unified species concept, under which species are defined as independently evolving metapopulation lineages (De Queiroz, 2007). This concept distinguishes between the ontological or primary concept and the operational criteria employed in species delimitation. The latter is done evaluating the acquisition of species properties (e.g., morphological diagnosability, reciprocal monophyly, reproductive isolation, or ecological differences; Sites Jr & Marshall, 2003; Knowles & Carstens, 2007; De Queiroz, 2007). There is a gray zone where different datasets and approaches would, eventually, provide conflicting schemes of species limits (De Queiroz, 1998; De Queiroz, 2007) due to lineages acquire their distinctive properties at different times and with varying order; in addition, some property may even not be acquired at all (e.g., two sister species may be morphologically indistinguishable; e.g., Bravo, Remsen Jr & Brumfield, 2014). For this reason, the integration of distinct datasets is the best way to proceed when aimed to establish species limits.

Patagonia comprises the southern end of continental South America and is a geographical term broadly used to encompass continental areas roughly south of 40°S. Four major biomes are represented in this region (Oyarzabal et al., 2018). Two forested areas are associated with the southern Andes; the Valdivian temperate rainforest to the north and the Magellan subpolar forest to the south. The Monte is a low shrubby steppe found in northeastern Patagonia. Finally, the Patagonian steppe is a large, open, and mostly arid area covered by herbaceous to shrubby steppes, which includes a more mesic area of grassland by the Strait of Magellan. There are ca. 84 living native species of mammals in Argentinean Patagonia and about 48 are terrestrial small mammals (<250 g), being 43 rodents, four marsupials, and one armadillo (Formoso et al., 2016).

The genus *Ctenomys* is endemic to the southern half of South America, occurring mostly in well-drained and friable soils, both at high and lowland areas, from southern Peru to The Island of Tierra del Fuego (Bidau, 2015). With about 68 recognized living species, this genus is the second most diverse of the order Rodentia, being exceeded only by *Rattus*, with 69 species (Burgin et al., 2018). Species of *Ctenomys* have mostly allopatric distributional ranges and are conservative in their morphology; in addition, several species differ in their chromosomal characters (Bidau, 2015). Despite that the systematics of this group has been intensively studied during the last five decades (e.g.,  Reig et al., 1992; Tiranti et al., 2005; Parada et al., 2011; Parada et al., 2012; Londoño Gaviria et al., 2019), our knowledge about the alpha taxonomy of *Ctenomys* is still far from complete (see a synthesis in Bidau, 2015). In fact, new candidate species are frequently identified (e.g.,  Parada et al., 2011; Caraballo & Rossi, 2017) and described (e.g., Freitas et al., 2012; Gardner, Salazar Bravo & Cook, 2014), at the time that some synonymies are also proposed (e.g.,  Teta, D’Elía & Opazo, 2020).

Six species of *Ctenomys* (i.e., *C. coyhaiquensis* Kelt & Gallardo, 1994; *C. fodax* Thomas, 1910; *C. haigi* Thomas, 1919; *C. magellanicus* Bennett, 1836 [including *C. colburni* Allen, 1903]; *C. sericeus* Allen, 1903; and *C. sociabilis* Pearson & Christie, 1985) are recognized in Patagonia south of 42°S. Of these, all but *C. sociabilis* are included in the *C. magellanicus* species group, a moderately diverse clade defined by Parada et al. (2011; see also Teta, D’Elía & Opazo, 2020) that distributes across open landscapes of Patagonia and Tierra del Fuego.

Besides the species descriptions, a handful of studies have focused on taxonomic aspects of Patagonian *Ctenomys*, most of which has been centered on western and southern populations currently assigned to *C. magellanicus* (e.g.,  Lizarralde et al., 2001; Teta, D’Elía & Opazo, 2020). In turn, the information on populations from eastern and northeastern portions of Patagonia is scarce. Thomas (1929) referred most samples from northeastern coastal Patagonia as *C. sericeus*, while most of the subsequent authors used an open nomenclature (e.g.,  Pardiñas et al., 2003; Udrizar Sauthier & Pardiñas, 2014). Almost a decade ago, Montes et al. (2001) and Bidau, Marti & Giménez (2002), based on karyological and molecular evidence, recognized at least three distinct forms occurring along the coastal region of Chubut, in northeastern Patagonia. At that time, these authors suggested the need of a careful review of some nominal forms from other Patagonian areas (e.g., *C. colburni*, *C. sericeus*), in order to clarify if those coastal lineages represent undescribed species or eastern representatives of so far known western distributed species.

In this study, we employed an integrative approach, analyzing mtDNA sequences and qualitative and quantitative morphological traits of skins and skulls, aimed to clarify the taxonomic status of eastern Patagonian populations of *Ctenomys*. We embrace the General Species Concept that states species are metapopulational lineages recognized by their emerging properties (e.g., monophyly, morphological diagnosability; De Queiroz, 2007). As a result of our analyses, three new species of *Ctenomys*, one comprised of two subspecies, are described and named. In addition, we revaluate the distinction of *C. coyhaiquensis*, from its sister species *C. sericeus*.

## Materials & Methods

No specimen was collected in the field in order to conduct this study. All studied specimens were already deposited in biological collections (see File S1).

*Sampling for the genetic and phylogenetic analyses*—Analyses are based on sequences of the cytochrome b gene (801 bp) retrieved from 65 specimens of the *C. magellanicus* species group collected at 25 localities and representing all but *C. fodax* known species of the group and the coastal forms earlier studied by Montes et al. (2001) and Bidau, Marti & Giménez (2002). In addition, sequences of specimens of *C. boliviensis*, *C. sociabilis*, *C. torquatus*, and *C. tucumanus*, representing other species groups of *Ctenomys* (Parada et al., 2011), were used to conform the outgroup. Sequenced specimens are listed in File S1. Sequences were retrieved from Genbank and generated in this work from specimens housed in Colección Felix de Azara (CFA-MA, Buenos Aires, Argentina). New sequences were gathered as in Teta, D’Elía & Opazo (2020) from pieces of skin of specimens collected between 20 and 40 years ago. DNA from these samples was extracted following the protocol of Velazco & Patterson (2013) and the cyt b gene was amplified in two fragments using primers MVZ05-oct439R and OCT406F-MVZ16. Amplicons were purified and sequenced by Macrogen Inc., Korea. New sequences were edited with Codon Code and deposited in Genbank. All accession numbers are provided in the File S1.

*Genetic and Phylogenetic analyses*—Sequences were aligned with Clustal as implemented in MEGA 6 (Tamura et al., 2013) using the default parameter values; the alignment was visually inspected to check for the presence of internal stop codons and reading frame shifts; no correction was needed. A gene tree was inferred via Bayesian inference (BI; Rannala & Yang, 1996) and Maximum Likelihood (ML; Felsenstein, 1981). The BI analysis was done with MrBayes 3.1 (Ronquist & Huelsenbeck, 2003). Two independent runs with 5 heated and 1 cold Markov chains each were run for 20 million generations, with trees sampled every 1,000 generations. The implemented model of nucleotide substitution (HKY + G) was selected using jModelTest (Darriba et al., 2012). Model parameters were estimated in Mr Bayes; base composition and HKY parameters assumed a Dirichlet process prior; all other parameters have uniform interval priors. By plotting log-likelihood values against generation time we checked convergence on stable log-likelihood values. The first 25% of the trees sampled were discarded as burn-in; remaining trees were used to compute a 50% majority rule consensus tree and to obtain posterior probability (PP) values for each clade. The ML analysis was conducted with RAxML-NG (Kozlov et al., 2019) as implemented online in the web service of the Swiss Institute of Bioinformatics (https://raxml-ng.vital-it.ch). Nodal support was evaluated via Bootstrap (BS); the sufficient number of replicates was determined automatically using the autoMRE bootstrap convergence test (Pattengale et al., 2009; see also Pattengale et al., 2010). Observed percentage of sequence divergence (p-distances) between pairs of haplotypes, as well as species was calculated with MEGA 6 ignoring sites with missing data.

**Figure 1:**
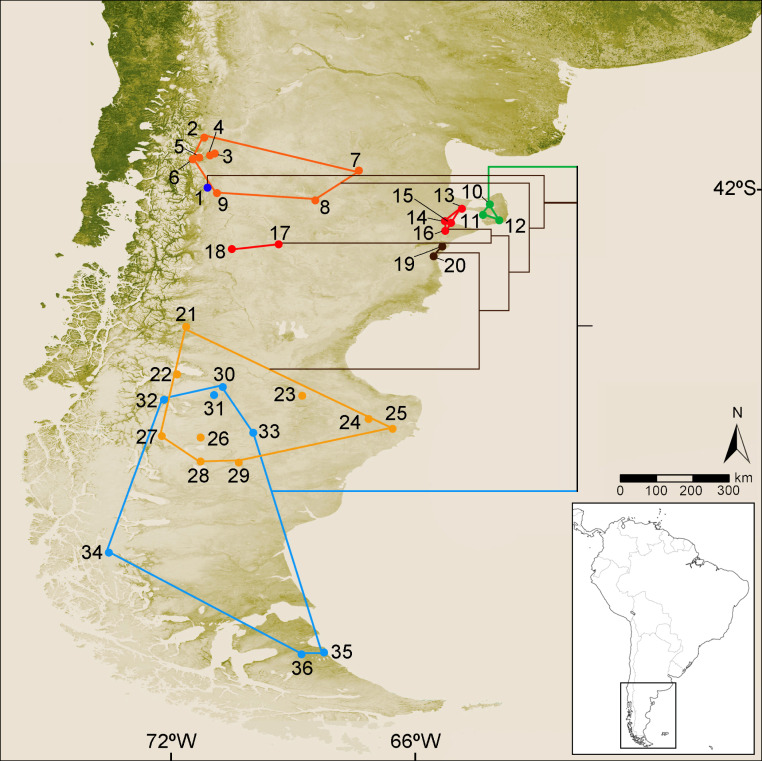
Collection localities of the specimens of *Ctenomys* analyzed in this study and simplified version of the cytochrome-b gene tree. Map of southern South America depicting the collection localities of the specimens of *Ctenomys* analyzed in this study. Locality data for each specimen is provided in File S1. Colors are as follow: blue dot, *C. haigi* s.s.; orange dots, *Ctenomys*. cf. *C. lentulus*; green dots, *C. bidaui* n. sp. (= PVC); red dots (localities 13–16), *C. c. contrerasi* n. subsp. (= NCR); red dots (localities 17–18), *C. c. navonae* n. subsp. (= WCC); brown dots, *C. thalesi* n. sp. (= SCR); yellow dots, *C. sericeus*; light blue dots, *C. magellanicus*. Superimposed to the map is depicted a simplified version of the cytochrome-b tree (see details in Fig. 2).

*Studied specimens in the morphological analyses*—Qualitative and quantitative morphological analyses were carried out on 75 adult specimens of *Ctenomys* collected at 19 localities in southern Argentina and Chile (Fig. 1); these specimens are housed in the following biological collections: Centro Nacional Patagónico (CNP, Chubut, Argentina); Field Museum of Natural History (FMNH, Chicago, US); Fundación de Historia Natural “Félix de Azara” (CFA, Buenos Aires, Argentina); Museo Argentino de Ciencias Naturales “Bernardino Rivadavia” (MACN-Ma, Buenos Aires, Argentina); Universidad Austral de Chile (UACh, Valdivia, Chile); U. S. National Museum of Natural History, Smithsonian Institution (USNM, Washington DC, US). Studied specimens and their collection localities are listed in the File S1.

*Anatomical descriptions and cranial measurements*—Terminology used to describe external and skull traits follows Langguth & Abella (1970), Contreras & Berry (1982), and Gardner, Salazar Bravo & Cook (2014). Fur coloration was described following the nomenclature of Ridgway (1912). Standard external measures, taken from field catalogs or specimen tags, include: head and body length (HBL); tail length (TL), hind foot length (including the claw) (HF); ear length (EL), and weight (W). Sixteen craniodental measurements were gathered from each specimen using a digital caliper to the nearest 0.01 mm and following the definitions provided by Contreras & Contreras (1984). Included measurements are: total length of the skull (TLS); condylo-incisive length (CIL); nasal length (NL); nasal width (NW); rostral width (RW); frontal length (FL); interorbital constriction (IOC); greatest zygomatic breadth (ZB); braincase breadth (BB); bimeatal breadth (BIB); mastoid breadth (MB); infraorbital foramen height (IFH); upper diastema length (DL); palatal length (PL); upper fourth premolar length (PM4L); and upper toothrow length (TRL).

*Morphological analyses*—Morphological comparisons among specimen samples were guided (e.g., composition of compared groups) by the results of the analyses of molecular data (see results below), geography, and current taxonomy. Patterns of variation among groups were assessed trough descriptive statistics (i.e., mean, minimum and maximum values, standard deviation) and multivariate statistical analyses. Employed statistical techniques consist of principal component (PCA) and discriminant function analyses (DFA). Principal components (PCs) were extracted from the variance–covariance matrix, after the log10-transformation of the original data (Strauss, 2010). DFA was used to determine a linear combination of morphometric traits that best defined those groups that were previously identified on the basis of qualitative morphological characters, chromosomes, and phylogenetic analyses of DNA sequences. Both sexes were pooled together in order to obtain larger samples in the statistical analyses (for a similar procedure, see Kelt & Gallardo, 1994).

Multivariate analysis conducted to test the differentiation between *C. coyhaiquensis* and *C. sericeus* considered five distinct geographical groups, two of them included within *C. coyhaiquensis*: Chile Chico [CHC (Fig. 1: locality (10)] and Coyhaique Alto [COA (9)]); and three among *C. sericeus*: western [WSC (14, 15)], central [CSC (16)], and eastern [ESC (11, 12)] Santa Cruz.

*New Zoological Taxonomic Names*—The electronic version of this article in Portable Document Format (PDF) will represent a published work according to the International Commission on Zoological Nomenclature (ICZN), and hence the new names contained in the electronic version are effectively published under that Code from the electronic edition alone. This published work and the nomenclatural acts it contains have been registered in ZooBank, the online registration system for the ICZN. The ZooBank LSIDs (Life Science Identifiers) can be resolved and the associated information viewed through any standard web browser by appending the LSID to the prefix http://zoobank.org/. The LSID for this publication is: urn:lsid:zoobank.org:pub:59FAE1BC-4C64-4071-A58D-F0E5E477B6E1. The online version of this work is archived and available from the following digital repositories: PeerJ, PubMed Central and CLOCKSS.

**Figure 2:**
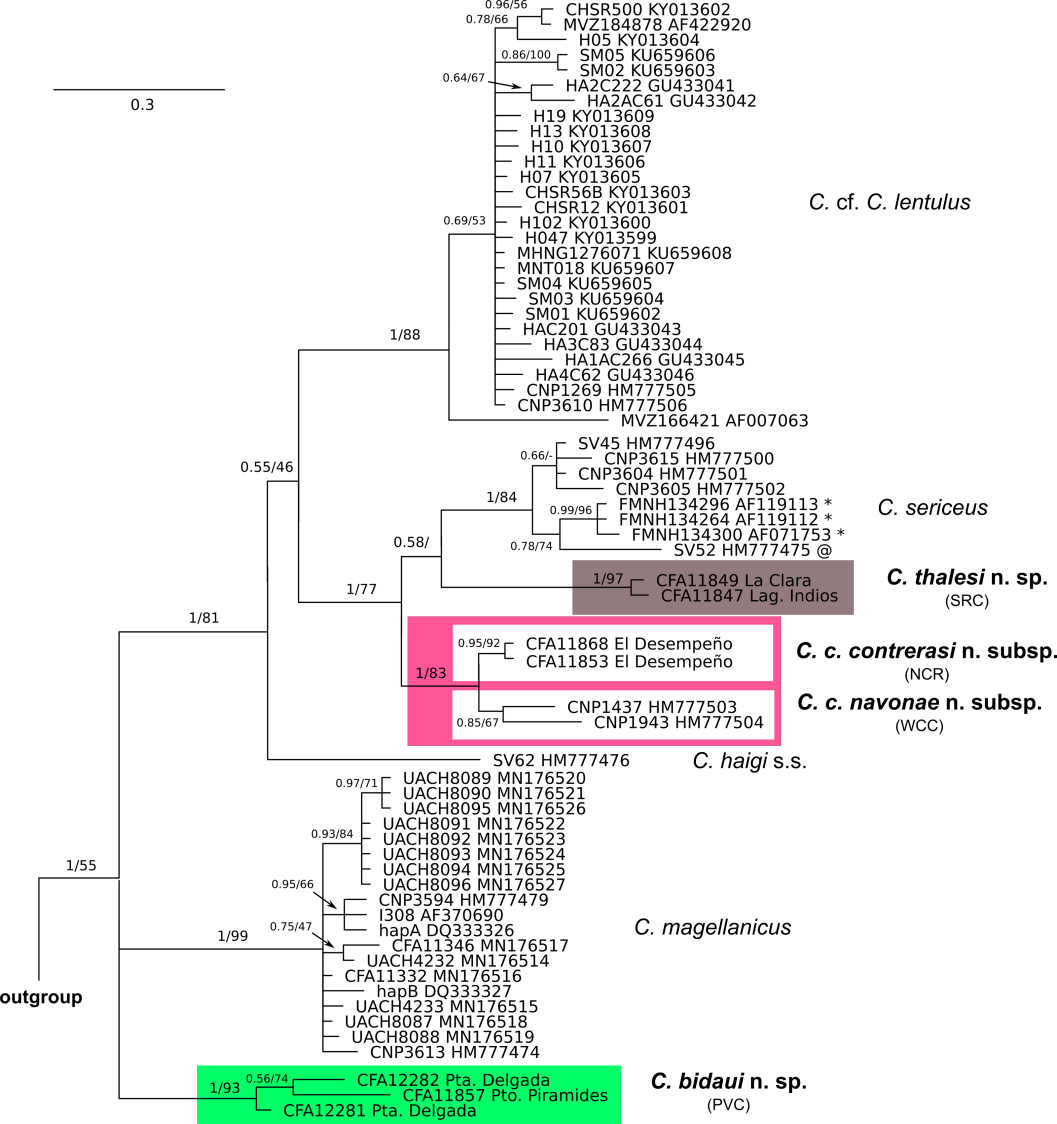
Cytochrome-b gene tree of haplotypes of specimens of the *Ctenomys magellanicus* species group. Majority rule consensus tree obtained from the Bayesian analysis of 65 cytochrome-b gene sequences of the *Ctenomys magellanicus* species group (sensu Parada et al., 2011). Numbers indicate posterior probability (left of the diagonal) and bootstrap (right of the diagonal) values of adjacent nodes; a missing value indicates that the given node has less than 50% of bootstrap support; a dash indicates that the given node was not recovered in the ML analysis. Terminal designations are the museum catalog and GenBank accession numbers, respectively. Haplotypes signaled with * were retrieved from specimens identified as *C. coyhaiquensis* before this study. The haplotype signaled with @ was retrieved from a specimen identified as *C. fodax* by Parada et al. (2011) and as *C*. sp. 1 by Teta, D’Elía & Opazo (2020). Locality data for each specimen is provided in File S1.

## Results

*Phylogenetic relationships*—The topologies resulted from the BI and ML analyses are mostly congruent; differences pertain to weakly supported relationships and will be noted below if of relevance. The *C. magellanicus* species group was recovered monophyletic (PP = 1; BS = 55). At the base of its clade in the BI tree there is a polytomy involving three lineages, one corresponding to *C. magellanicus* (PP = 1; BS = 99), other to a clade (PP = 1; BS = 93) composed by haplotypes collected at Península de Valdés, Chubut (referred as PVC), and the other to a large clade (PP =1; BS = 81) composed by all other haplotypes of the *C. magellanicus* species group (Fig. 2). In the ML tree the latter two lineages appear sister to each other in a weakly supported clade (BS = 50). Within the latter, haplotypes currently assigned to *C. haigi* do not form a monophyletic group, falling into two main lineages; one of these is composed by a single haplotype recovered from a topotype of the species, while the other corresponds to a widely distributed clade (PP = 1; BS = 88), including the general area of the type locality (“Pilcañeu, Upper Rio Negro…1,400 m”. Thomas, 1919: 211) of *C. lentulus* Thomas, 1919, a nominal form associated with *C. haigi* (Fig. 2). Haplotypes of *C. coyhaiquensis* form a clade (PP = 0.99; BS = 96) that is sister, in a weakly supported relationship (PP = 0.78; BS = 74), to a lineage composed by a single haplotype recovered from a specimen of *Ctenomys* referred as *C. fodax* in previous studies (e.g., Parada et al., 2011) but which according to Teta, D’Elía & Opazo (2020) does not belong to *fodax* but to an undetermined, not necessarily new, form allied to *sericeus* (*C*. aff. *C. sericeus*). In the BI tree haplotypes of *C. sericeus* form a weakly supported group (PP = 0.66) that is sister (PP = 1) to the *C. coyhaiquensis-C*. aff. *C. sericeus* clade (Fig. 2). In the ML tree haplotypes of *C. sericeus* form a paraphyletic group (BS = 84) to the clade form by haplotypes of *C. coyhaiquensis* and *C.* aff. *C. sericeus.* Subsequently, the clade including *C. sericeus*, *C. coyhaiquensis*, and *C*. aff. *C. sericeus* is sister, although with a very low support (PP = 0.55; BS < 50), to a clade (PP = 1; BS = 97) encompassing those samples from northeastern Chubut, south of the Chubut River (SCR) (Fig. 2). Haplotypes recovered from specimens collected at northeastern Chubut north of the Chubut River (NCR), form a clade (PP = 0.95; BS = 92) sister (PP = 1; BS = 83) to the clade (PP = 0.85; BS = 67) formed by haplotypes from west-central Chubut (WCC) (Fig. 2). Observed genetic variation within and among the main clades mentioned above are shown in Table 1; pairwise comparisons range from 0.8% for the pair NCR vs WCC to 5.2 for the pair SCR vs *C. magellanicus*.

*Morphological variation*—Qualitative morphological traits that characterized each main lineage are discussed below, on each taxonomic account (see a summary on Table 2 and raw measurement values in File S2). Quantitative morphological variation is discussed in the following paragraphs of this section.

**Figure 3:**
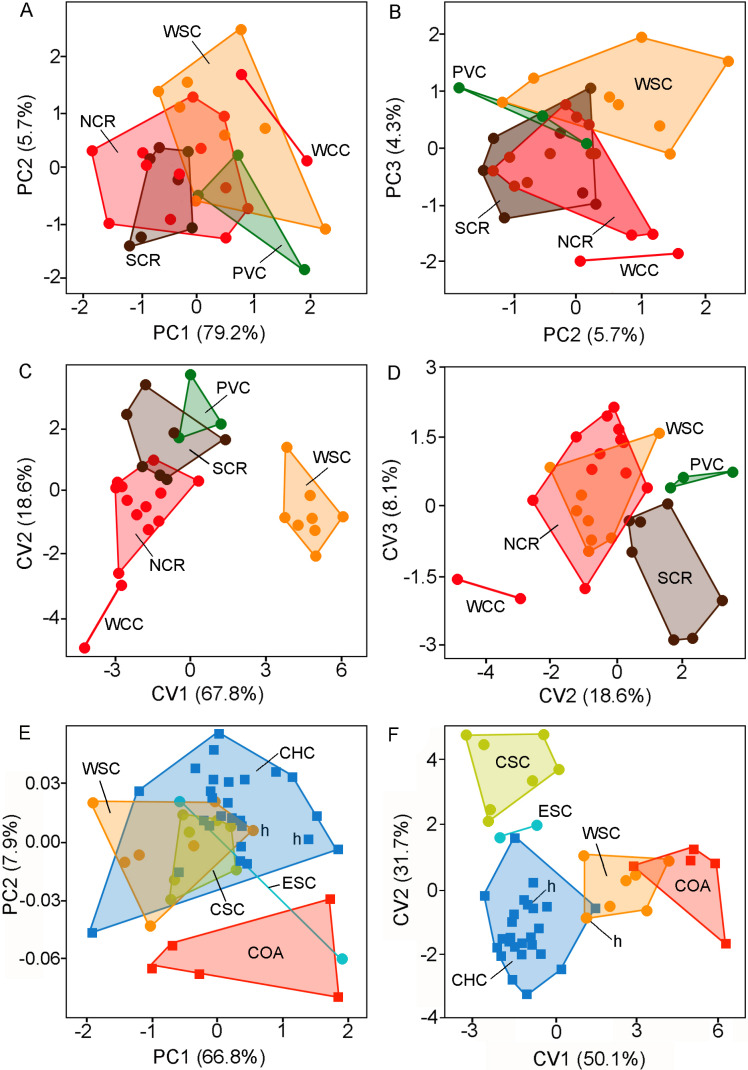
Individual scores (principal components and canonical variates) of adult specimens of *Ctenomys* from Patagonia. Individual scores of adult specimens of *Ctenomys* from Patagonia (*n* = 32) for: (A) principal components 1 and 2; (B) principal components 2 and 3; (C) canonical variates 1 and 2; and (D) canonical variates 2 and 3, extracted from five taxonomical group discriminant function analysis (i.e., NCR, PVC, SCR, WCC, and WSC; colors are as in Figs. 1 and 2); and individual scores of adult specimens of *Ctenomys sericeus* (*n* = 16) and *C. coyhaiquensis* (*n* = 31) for:(E) principal components 1 and 2, and (F) canonical variates 1 and 2 extracted from five geographical group discriminant function analyses (i.e., COA, CHC, CSC, ESC, and WSC). In (E) and (F), the holotypes of *C. sericeus* and *C. coyhaiquensis* are depicted with an “h”. See the text and Fig. 1 for the acronyms of the geographical groups.

The PCA revealed that all variables were positively correlated with the 1st principal component (PC1 79.2%). This situation suggests that PC1 summarizes mainly latent size variation. The plot of individual scores shows a moderate overlap of individuals from different forms or putative species in relation to the PC1 (Fig. 3A) being the holotype and topotypes of *C. sericeus* (= WSC) and the forms from PVC and WCC on average larger than those from NCR and SRC (see also Table 3). The distribution along the 2nd principal component (PC2 5.7%, Figs. 3A–3B; Table 4) contributes to separate the forms PVC and SCR from WCC. The DFA revealed two major morphometric clusters along the 1st discriminant axe (Fig. 3C; Table 4), which summarize 67.8% of the total variance. The 1st group is composed exclusively by the holotype and topotypes of *C. sericeus* (WSC), while the 2nd cluster comprises the four remaining forms (i.e., PVC, NCR, SCR, and WCC). These four forms are dispersed along the 2nd axis, which contributes with 18.6% of the total variance. Plot of individual scores along the 2nd and 3rd axes helped to discriminate between PVC, SCR, and WCC from the holotype and topotypes of *C. sericeus* (WSC) and NCR (Fig. 3D).

The PCA employed to assess the differentiation between the nominal forms *coyhaiquensis* and *sericeus* also shown that all variables were positively correlated with the 1st principal component (PC1 66.8%; see also Tables 1S and 2S, Data S1). The plot of individual scores along the two first PCs shows a large overlap among individuals from four different geographical groups, including the topotypical samples of the two nominal forms (Fig. 3E; Table 2S, Data S1). The PC2 (7.9%) contributes to separate the individuals from Coyhaique Alto from the remaining samples (although one specimen from eastern Santa Cruz was placed near this group). The DFA shows a good discrimination along the two first discriminant axes (81.8% of the variance; Fig. 3F; Table 2S, Data S1) of the five groups defined a priori. Samples referred to *C*. *coyhaiquensis* and *C. sericeus* were mixed in the multivariate space defined by the two first axes, with those individuals corresponding to topotypical samples (including the holotypes) closely placed (Fig. 3F).

*Taxonomy*—Despite that some examined sample sizes are small, we consider that when taken together, the karyological, morphological, and molecular data provide evidence that the current taxonomic scheme does not reflect the real species diversity of the *C. magellanicus* species group. At the time that some currently recognized species are not distinct (i.e., *C. coyhaiquensis*), there are lineages of species level (i.e., those here referred as the forms PVC, NCR, SCR, and WCC) distinct from *C. fodax*, *C. haigi*, *C. magellanicus* (sensu Teta, D’Elía & Opazo, 2020), and *C. sericeus* (including *C. coyhaiquensis*). Given the low genetic distance observed between two of these groups (NCR and WCC), the low support for each of these groups in the gene trees and that together they form a strongly supported clade, even when they morphologically differ, we consider them as representing one species. As no name is available for any of the three forms here recognized as distinct species (one with two subspecies), we name and describe them below. As *C. sericeus* is here redefined, we also include a brief account for this species.

**Table 1:** Percentage of average genetic variation (p-distances), based on cytochrome-b (Cytb) sequence data, observed within and between pairs of taxa of *Ctenomys*. Sample sizes are given between parentheses.

	intra	*C. bidaui* n. sp. (PVC)	*C. thalesi* n. sp. (SRC)	*C. contrerasi* n. subsp. (NCR)	*C. navonae* n. subsp. (WCC)	*C. sericeus*	*C. haigi s.s.*	*C. cf. C. lentulus*
*C. bidaui* n. sp. (3)	0.9							
*C. thalesi* n. sp. (2)	0	4.2						
*C. contrerasi* n. subsp. (2)	0	4.0	2.0					
*C. navonae* n. subsp. (2)	1.0	4.4	2.6	0.8				
*C. sericeus* (8)	0.7	3.6	2.6	2.2	2.5			
*C. haigi s.s.* (1)	n/c	4.2	5.0	3.4	3.8	3.9		
*C. cf. C. lentulus* (28)	0.7	4.4	3.7	3.2	3.4	3.3	3.6	
*C. magellanicus* (19)	0.4	3.4	5.2	4.7	5.2	4.6	4.4	4.8

**Table 2:** Selected traits compared among *Ctenomys bidaui* n. sp., *Ctenomys contrerasi contrerasi* n. subsp., *Ctenomys contrerasi navonae* n. subsp., *Ctenomys thalesi* n. sp., and *Ctenomys sericeus*.

	***C. bidaui*n. sp.** (PVC)	***C. c. contrerasi*n. subsp.** (NCR)	***C. c. navonae*n. subsp.** (WCC)	***C.thalesi*n. sp.** (SRC)	***C. sericeus***
Size	medium	small	medium	small	small
**Premaxillo-frontal suture**	level with naso-frontal suture	sligthly behind the naso-frontal suture	well behind the naso-frontal suture	sligthly behind the naso-frontal suture	well behind the naso-frontal suture
**Interparietal**	broad and short	fused	fused	fused	fused or indistinct
**Zygomatic arches**	Robust	thin	moderately robust	thin	moderately robust
**Incisive foramina**	moderately short and broad	moderately long and narrow	moderately long and narrow	moderately short and narrow	moderately short and broad
**Interpremaxillary foramen**	large	small	large	small to absent	small to absent
**Auditory bullae**	inflated and ovate	inflated and pyriform	inflated and pyriform	inflated and pyriform	inflated and pyriform
**Paraoccipital processes**	quadrate	hook-shaped	hook-shaped	fan-shaped	hook-shaped
**2N; FN**	48; 72	38; 42 or 52	unknown	28; 40	28-30; 44-46

**Table:** 

**Figure 4:**
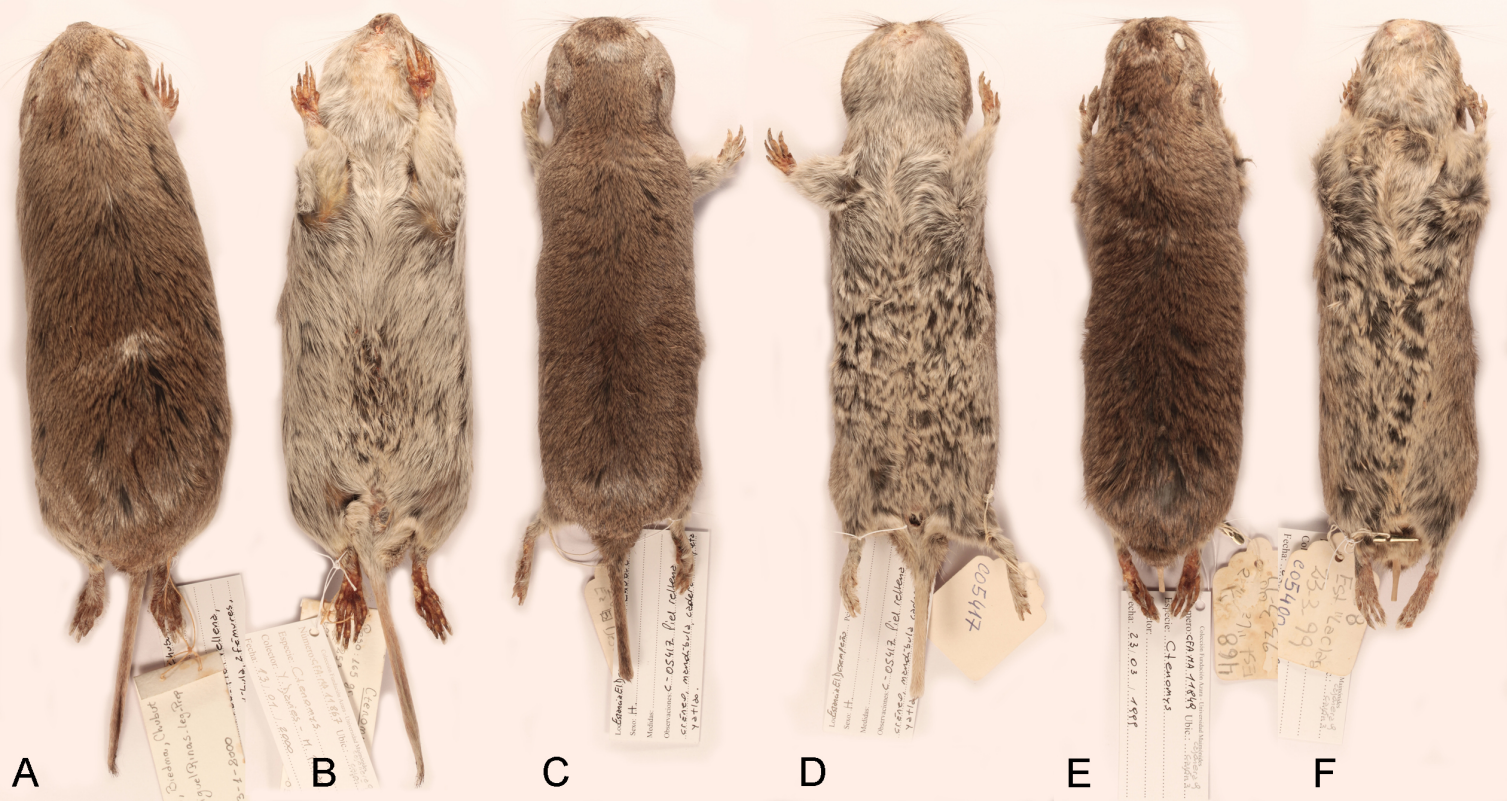
Skins of the holotypes of *Ctenomys bidaui* n. sp., *Ctenomys contrerasi* n. sp., and *Ctenomys thalesi* n. sp. Dorsal (A, C, E) and ventral (B, D, F) views of the skins of the holotypes of *Ctenomys bidaui* n. sp. (A, B; CFA-MA 11867), *C. contrerasi* n. sp. (C, D; CFA-MA 11853), and *C. thalesi* n. sp. (E, F; CFA-MA 11849).

**Figure 5:**
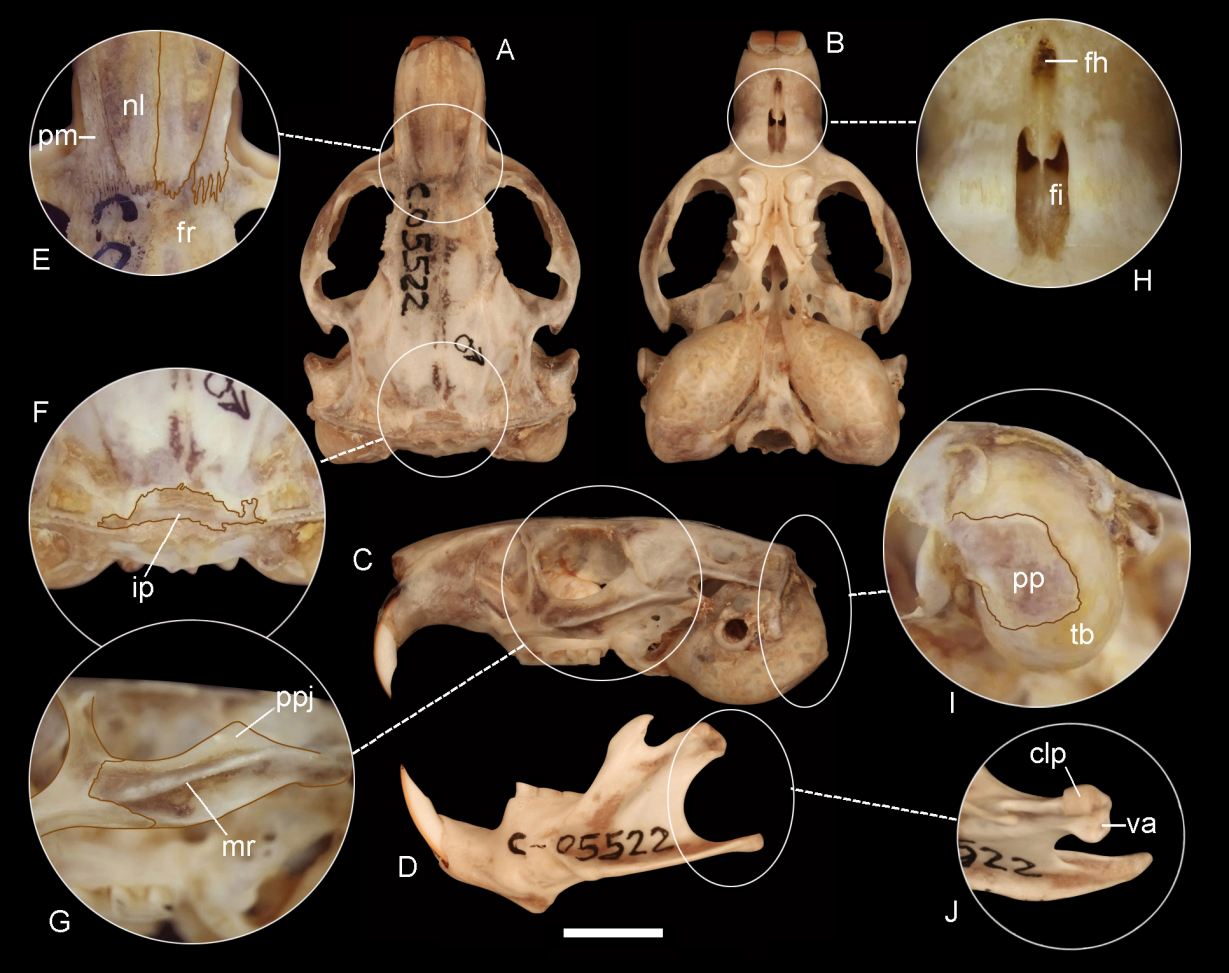
Holotype of *Ctenomys bidaui* n. sp. (CFA-MA 11867). Dorsal (A), ventral (B) and lateral (C) views of the skull and labial view of the mandible (D) and selected cranial traits (E–J) of the holotype of *Ctenomys bidaui* n. sp. (CFA-MA 11867). E: nasals (nl) and premaxillae (pm); F: interparietal (ip); G; lateral view of the zygomatic arch; H: incisive (fi) and interpremaxillary (fh) foramina; I: tympanic bullae (tb) in posterolateral view; J: condyloid process (clp) of the mandible in dorsal view. Other abbreviations: fr, frontals; mr, masseteric ridge; pp, paraoccipital process; ppj, postorbital process of jugal; va, ventrolateral apophysis of the postcondyloid process. Scale = 5 mm.

***Ctenomys bidaui*** n. sp.
urn:lsid:zoobank.org:act:B90C9672-525E-4E00-BA69-0EE58F4159E8
Bidau’s tuco-tuco
Tuco-Tuco de Bidau
PVC above
(Figs. 4 and 5)

*Ctenomys* aff. *colburni*: Daciuk (1974: 29).

**Table 3:** Results of principal components analyses (first, second, and third columns) and discriminant function analysis (fourth, fifth, and sixth columns) performed on four species of adult specimens of *Ctenomys* (*n* = 32). See ‘Materials and Methods’ for explanation of abbreviations.

	**PC 1**	**PC 2**	**PC 3**	**CV1**	**CV2**	**CV3**
**TLS**	0.2441	0.0736	0.0121	194.190	−199.850	91.295
**CIL**	0.2593	0.1022	0.0488	−211.330	91.856	−58.718
**NL**	0.2211	0.0570	−0.2927	−25.375	39.623	11.656
**NW**	0.3208	−0.3105	−0.2616	−17.858	0.356	−67.057
**FL**	0.1013	0.4374	0.5292	11.691	80.611	−33.431
**RW**	0.3026	−0.1694	−0.0968	19.629	24.123	−31.217
**ZB**	0.2361	−0.2113	0.0797	−58.422	50.557	54.675
**IOC**	0.2088	−0.4623	0.1863	−40.370	15.997	17.102
**BB**	0.0798	−0.2447	0.0292	−25.949	−23.724	−28.129
**BIB**	0.2281	−0.0260	−0.1856	−73.318	−44.216	36.949
**MB**	0.2019	−0.0618	0.0140	61.675	81.031	55.401
**IFH**	0.2484	−0.0271	0.3688	61.711	−27.191	20.447
**DL**	0.3937	0.1413	0.2728	89.420	−88.507	−32.668
**PM4L**	0.2617	0.5495	−0.3796	−0.902	23.224	57.166
**PL**	0.3240	0.0588	0.1391	−15.295	−25.929	−53.192
**TRL**	0.1758	0.1253	−0.3217	−32.149	−25.869	−32.666
Eigenvalue	0.0170	0.0013	0.0009	9.54	2.62	1.14
% variance	79.29	5.74	4.34	67.77	18.61	8.11

**Table 4:** Summary statistics (mean, SD, range) of cranial measurements (in mm) of adult samples (n) of the four new taxa of *Ctenomys* described here. See Materials and Methods for abbreviations For individual measurements, see File S2.

******	***C. bidaui*n. sp.**					***C. c. contrerasi*n. subsp.**					***C. c. navonae*n. subsp.**					***C. thalesi*n. sp.**				
****	**n**	**Mean**	***SD***	**Min.**	**Max.**	**n**	**Mean**	***SD***	**Min.**	**Max.**	**n**	**Mean**	***SD***	**Min.**	**Max.**	**n**	**Mean**	***SD***	**Min.**	**Max.**
**TOTL**	5	232.6	21.4	205	253	10	196.5	17.3	165	224	2	–	–	220	232	8	204.2	13.4	183	221
**TAIL**	5	64.3	9.7	53	73.2	10	59.7	4.5	49.4	65.3	2	–	–	68	68	8	62.3	3.1	57.4	66.2
**HFL**	5	32.1	1.5	30.2	34.2	10	26.8	1.6	23.6	28.7	2	–	–	29	32	8	26.9	1.6	23.7	29.1
**EAR**	5	7.1	0.9	6.3	8.3	10	6.9	0.6	5.9	7.6	2	–	–	6	7	8	6.6	0.4	6	7
**W**	5	132.8	24.8	105	165	11	77.1	26.2	43	117	2	–	–	118	146	8	71.9	15.9	43	96
**TLS**	3	38.61	2.63	36.06	41.31	12	36.03	2.29	32.12	39.28	2	40.70	1.72	39.48	41.91	7	34.58	1.43	32.47	36.55
**CIL**	3	37.25	2.91	34.42	40.24	12	34.23	2.39	30.12	37.34	2	38.71	1.94	37.34	40.08	7	32.83	1.55	30.83	35.11
**NL**	3	13.10	0.93	12.04	13.78	12	12.44	1.00	10.60	13.84	2	14.29	0.08	14.23	14.34	7	11.81	0.60	11.03	12.71
**NW**	3	5.79	0.95	5.13	6.88	12	5.04	0.48	4.21	5.85	2	6.19	0.18	6.06	6.31	7	5.13	0.29	4.90	5.78
**FL**	3	11.58	0.35	11.23	11.93	12	10.89	0.42	10.19	11.50	2	11.50	0.88	10.87	12.12	7	10.84	0.59	10.18	11.84
**RW**	3	8.39	0.36	8.18	8.81	12	7.39	0.59	6.32	8.01	2	8.60	1.32	7.66	9.53	7	7.27	0.32	6.89	7.85
**ZB**	3	23.65	2.50	21.61	26.44	12	21.32	1.36	19.17	23.90	2	23.24	1.29	22.33	24.15	7	20.57	0.63	19.41	21.29
**IOB**	3	7.59	0.55	7.24	8.22	12	6.86	0.55	6.18	7.94	2	7.19	1.03	6.46	7.91	7	6.82	0.19	6.65	7.13
**BB**	3	15.80	0.79	15.30	16.71	12	15.30	0.23	14.95	15.64	2	15.85	1.04	15.11	16.58	7	15.25	0.35	14.71	15.67
**BIB**	3	24.34	1.46	23.16	25.98	12	22.62	1.59	20.20	24.51	2	25.96	0.16	25.84	26.07	7	21.64	0.60	20.89	22.71
**MB**	3	23.47	1.82	22.09	25.54	12	21.23	1.18	19.65	22.70	2	23.08	0.88	22.45	23.70	7	21.01	0.61	20.07	21.86
**IFH**	3	7.37	0.84	6.69	8.31	12	6.52	0.42	5.94	7.17	2	7.11	0.52	6.74	7.47	7	6.38	0.36	5.77	6.75
**DL**	3	9.84	1.03	8.69	10.67	12	8.62	0.91	6.92	10.06	2	10.16	1.13	9.36	10.96	7	8.16	0.53	7.47	8.91
**PL**	3	16.26	1.77	14.49	18.02	12	14.44	1.20	12.22	16.11	2	16.72	1.36	15.76	17.68	7	14.00	0.82	13.05	15.11
**PM4L**	3	2.90	0.06	2.84	2.95	12	2.74	0.30	2.24	3.23	2	3.43	0.29	3.22	3.63	7	2.68	0.15	2.41	2.85
**TRL**	3	7.45	0.46	6.93	7.79	12	7.37	0.42	6.79	8.14	2	8.48	0.37	8.22	8.74	7	7.12	0.22	6.87	7.41

*Ctenomys* [sp.]: Montes et al. (2001: 79).

*Ctenomys* [sp.]:Bidau, Marti & Giménez (2002: 64).

*Ctenomys* sp.: D’Agostino, Udrizar Sauthier & Nabte (2017: 181).

*Holotype*—CFA-MA 11867 (previously referred as C-05522 in the personal collection of Julio R. Contreras), adult male; skin, skull and partial skeleton collected by Yolanda E. Davies with date 13 January 2000.

*Type locality*—Argentina: Chubut, Biedma, Puerto Pirámides (-42.57, -64.28) (Fig. 1: locality 2).

*Measurements of the holotype (in mm)*—TOTL, 253; TAIL, 73; HFL, 34.2; EAR, 6.6; TLS, 41.31; CIL, 40.24; NL, 13.78; NW, 6.88; FL, 11.93; RW, 8.81; ZB, 26.44; IOB, 8.22; BB, 16.71; BIB, 25.98; MB, 25.54; IFH, 8.31; DL, 10.77; PL, 18.02; PM4L, 2.95; TRL, 7.62. Weight, 165 g.

*Paratypes*—CFA-MA 11857 (C-05523), adult female; skin, skull and partial skeleton; and CFA-MA 11865 (C-05524), adult male; skin, skull, and partial skeleton. Both specimens were collected by Yolanda E. Davies with date 13 January 2000 at the species type locality.

*Other examined specimens*—CFA-MA 12281, CFA-MA 12282, MACN-Ma 16395 (see File S1 for additional details).

*Morphological diagnosis*—A medium-sized tuco-tuco of the *C*. *magellanicus* species group with moderately differentiated dorsal and ventral colorations; dorsum Light Brownish Olive to Brownish Olive; venter Pale Olive Buff with Gray colored basal hairs. Skull strongly built; interorbital processes of frontals slightly developed; zygomatic arches robust; premaxillo-frontal suture at the level of the naso-frontal suture; interparietal broad and short; incisive foramina moderately short and broad, recessed in a common fossa of straight outer borders and incompletely separated by a bony septum; interpremaxillary foramen large; auditory bullae inflated and ovate.

*Sperm type*—Asymmetric (Bidau, Marti & Giménez, 2002).

*Karyotype*—Specimens from Punta Delgada (Fig. 1: locality 3) presented a karyotype with 2N = 48 and FN = 72, with 5 autosomic pairs acrocentric, 6 submetacentric, 12 metacentric, and 10 telocentric; X metacentric, Y acrocentric (Bidau, Marti & Giménez, 2002).

*Morphological description*—Pelage dense, fine, and soft, about 10–12 mm long over back and rump; dorsum with fur ranging from Light Brownish Olive to Brownish Olive; individual hairs Dark Neutral Gray colored, except for the distal tips, which are lighter. Color of ventral pelage Pale Olive Buff; individual hairs Dark Gray basally, with superficial wash of White to Isabella. Fur of fore and hind limbs colored like dorsum and rump, except internal sides which are Pale Olive Buff. Top of manus and feet covered with whitish hairs. Mystacial vibrissae surpassing the dorsal edge of the pinnae when laid back alongside of head; superciliary vibrissae sparse, extending to the base of the pinnae when laid back alongside of head. Ears sparsely covered with short, brownish hairs. Pes broad, all digits with ungueal tufts of stiff bristles, and strong claws. Tail short, slightly darker above than below and sparsely covered by short hairs; its distal third is covered by a dorsal fringe of Dark Brown, longer hairs (Figs. 4A–4B). Skull strongly built; interorbital region with posteriorly divergent outer margins; zygomatic arches broad and nearly rounded in dorsal view; auditory bullae inflated and ovate, with salient auditory tubes (Figs. 5A–5D). Nasals short, broadest in their anterior third, and with nearly straight lateral margins. Premaxillary clearly visible when viewed from dorsal aspect, with the premaxillo-frontal suture at the level of the naso-frontal suture (Fig. 5E). Interorbital processes of frontals slightly developed. Interparietal broad and short (Fig. 5F). Temporal ridges moderately expressed. Supraoccipital crest strongly developed in adults. Zygomatic arches robust, with moderately developed postorbital process of jugal and a conspicuous zygomatic depression; mandibular processes of jugal not evident to moderately expressed (Fig. 5G). Incisive foramina moderately short and broad, recessed in a common fossa of straight outer borders, and incompletely separated by a bony septum (Fig. 5H). Interpremaxillary foramen large (Fig. 5H). Palatal bridge with two major palatine foramina at about level of M1. Mesopterygoid fossae “V” shape, reaching anteriorly the posterior portion of M2. Posteropalatal pits present and small, placed behind the M3. Alisphenoid-presphenoid bridge flat and narrow; bony roof of mesopterygoid fossa with two large sphenopalatine vacuities. Buccinator-masticatory foramen divided into two small foramina by a bony strut. Paraoccipital processes well developed, broad and nearly quadrate to round in outline (Fig. 5I). Upper incisors large, robust, and orthodont; frontal enamel surface Orange (Fig. 1SA-B, on Data S2). Maxillary tooth rows slightly divergent posteriorly. Mandible robust (Fig. 5C), with coronoid process falciform, and strongly angled backwards; condyloid process robust; bearing a well-developed articulation flange (Fig. 5J; Fig. 2SA, on Data S2). Descriptive statistics for external and cranial measurements are provided on Table 4.

*Distribution*—Known only from three localities near coastal areas of Península de Valdés, Chubut, Argentina (Fig. 1). Possibly, also correspond to this species the late Holocene fossil remains referred by Udrizar Sauthier & D’Agostino (2017) from this same general area.

*Etymology*—We named this species in honor of the late Claudio J. Bidau (1953-2018), an Argentinian biologist with an extensive and varied scientific production, of which an important fraction is aimed to elucidate the complex evolutionary history of the genus *Ctenomys*. Claudio was a much-appreciated member of the South American community of mammalogists where he is well remembered. The species name is a patronym in the genitive singular.

**Figure 6:**
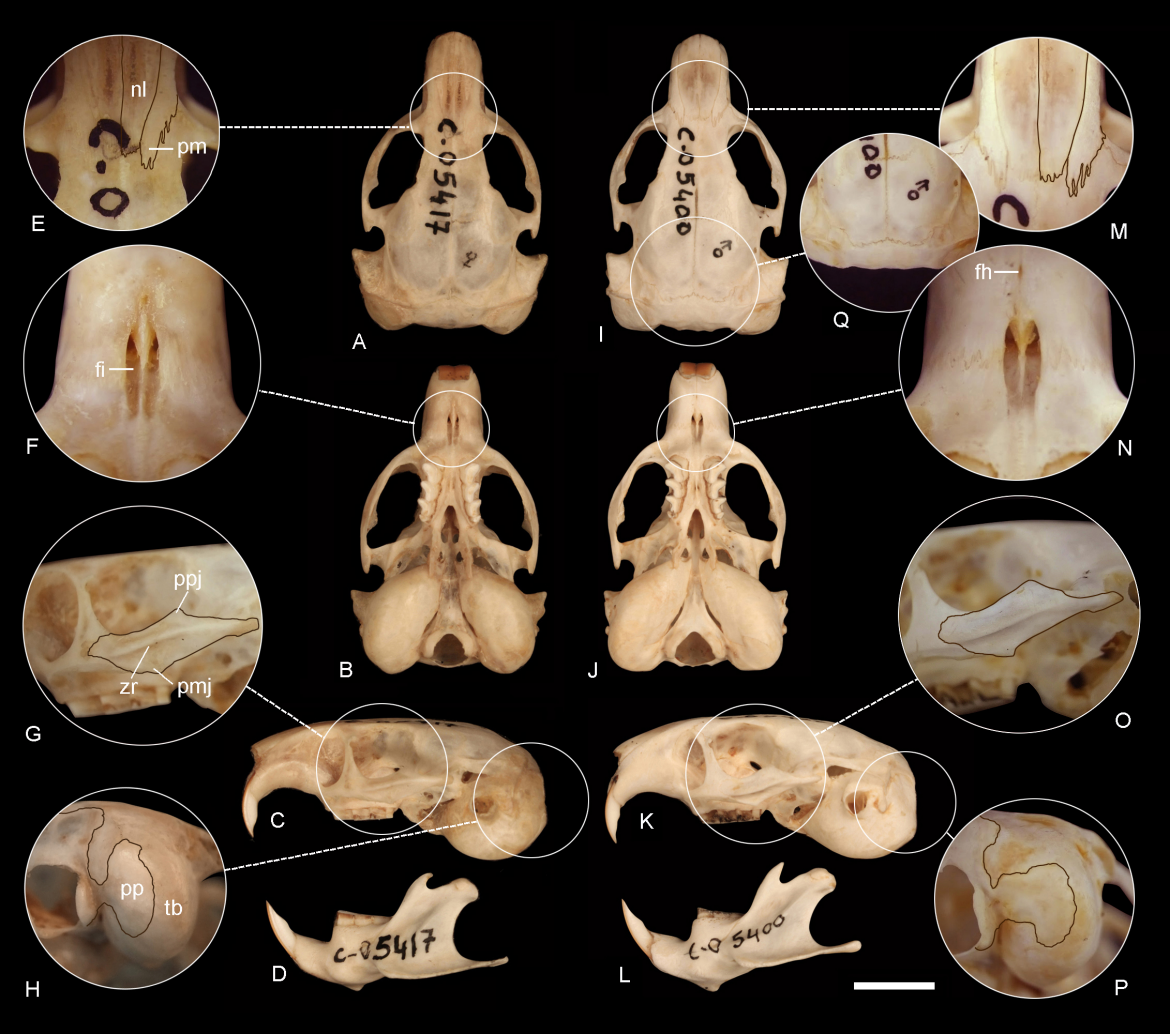
Comparison of the holotypes of *C tenomys contrerasi* n. sp. and *Ctenomys thalesi* n. sp. Dorsal (A, I), ventral (B, J) and lateral (C, K) views of the skull and labial views of the mandibles (D, L) and selected cranial traits (E–H, M–Q) of the holotypes of *Ctenomys contrerasi* n. sp. (CFA-MA 11853) and *C. thalesi* n. sp. (CFA-MA 11849). E, M: nasals (nl) and premaxillae (pm); F, N: incisive (fi) and interpremaxillary (fh) foramina; G, O: lateral views of the zygomatic arches; H, P: tympanic bullae (tb) in posterolateral view; Q: posterior portion of the braincase, showing the absence of interparietal. Other abbreviations: bs, bony septum; mr, masseteric ridge; pp, paraoccipital process; ppj, postorbital process of jugal; pmj, mandibular process of jugal. Scale = 5 mm.

**Figure 7:**
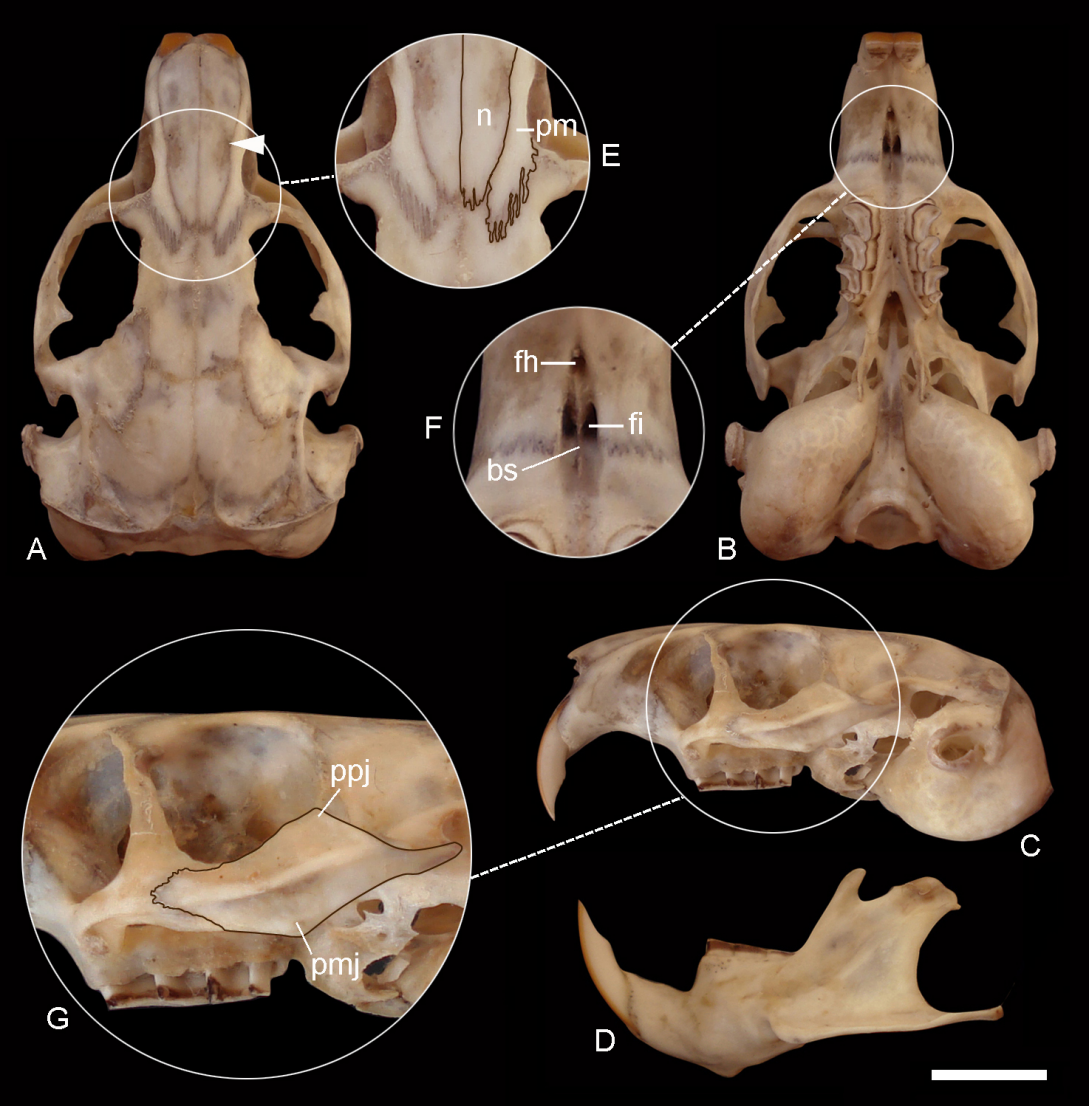
Holotype of *Ctenomys contrerasi navonae.* n. subsp. (CNP 1043). Dorsal (A), ventral (B) and lateral (C) views of the skull and labial view of the mandible (D) and selected cranial traits (E–G) of the holotype of *Ctenomys contrerasi navonae* n. subsp. (CNP 1043). E: nasals (n) and premaxillae (pm); F: incisive (fi) and interpremaxillary (fh) foramina; G: lateral view of the zygomatic arch. Other abbreviations: bs, bony septum; mr, masseteric ridge; ppj, postorbital process of jugal; pmj, mandibular process of jugal. Scale = 5 mm.

*Morphological comparisons*—While *C. bidaui* n. sp. has the premaxillo-frontal suture placed at the level of the naso-frontal suture, in most species of the *C*. *magellanicus* species group, including *C. sericeus* and the two new species described below, the premaxillo-frontal suture is placed behind the naso-frontal suture (Table 2; see Figs. 6 and 7). The absence of a bony septum between the incisive foramina and the sape and size of the paraoccipital process differentiate *C. bidaui* n. sp. from *C. sericeus* and the new species described below (for detailed comparison, see also Figs. 3S and 4S on Data S2). In addition, the diploid complement of *C. bidaui* is different from those of *C. sericeus* and two of the three new species described in this contribution for which karyological data is available (Table 2). This species differs from *C. haigi* (2n = 50) by having a different diploid complement and a much lighter coloration. *Ctenomys bidaui* n. sp. is conspicuously smaller than *C. fodax* (TLS ∼53 mm) and *C. magellanicus* (TLS ∼49 mm) and has a different diploid complement from the latter. Pairwise genetic distances between Bidau’s tuco-tuco and other species of *Ctenomys* range from 3.4 to 4.4% (Table 1).

*Natural history*—Mostly unknown; all three recording localities of Bidau’s tuco-tuco lay at the ecotone between the Monte and Patagonian phytogeographical provinces. The entire territory of Península de Valdés is placed within the unit “Estepa arbustiva ecotonal con *Chuquiraga avellanedae*” as delimited by Oyarzabal et al. (2018). The landscape corresponds to a plain with two depressed saline areas and sandy dunes along its southern border. The vegetation is dominated by low shrubs (0.5–1.5 m), such as *Chuquiraga avellanedae*, *C. erinacea*, and *Condalia microphylla*, intermixed with other low shrubs (e.g., *Brachyclados megalanthus*, *Lycium chilense*, *Schinus polygamus*, *Prosopidastrum globosum*, and *Larrea nitida*), and some hard grasses, such as *Nassella tenuis*, *Nassella longiglumis*, *Pappostipa speciosa*, *Piptochaetium napostaense*, and *Poa ligularis*. Sand dunes are characterized by gramineous steppes with *Sporobolus rigens*, *Nassella tenuis*, *Panicum urvilleanum*, *Poa lanuginose*, and *Piptochaetium napostaense*; total cover varies between 60% to 80% (Oyarzabal et al., 2018). D’Agostino, Udrizar Sauthier & Nabte (2017) provided some notes on the distribution and abundance of this tuco-tuco at Reserva de Vida Silvestre San Pablo de Valdés, 6 km SE Puerto Pirámides.

**Table:** 

***Ctenomys contrerasi*** n. sp.
urn:lsid:zoobank.org:act:14D8E298-5175-4688-A1EC-105F81163132
Contreras’tuco-tuco
Tuco-Tuco de Contreras

*Ctenomys* [sp.]: Montes et al. (2001: 79).

*Ctenomys* [sp.]: Bidau, Marti & Giménez (2002: 64).

*Ctenomys* sp.: Nabte, Saba & Monjeau (2009: 115).

*Ctenomys* sp. 4: Parada et al. (2011: 682).

*Ctenomys* sp. 5: Parada et al. (2011: 682).

*Holotype*—CFA-MA 11853 (C-05417), adult female; skin, skull and partial skeleton collected on 28 March 1999 by Mabel D. Giménez, Claudio J. Bidau, Dardo A. Marti, and Martín A. Montes (field number 485). A partial (801 bp) DNA sequence of the cytochrome- b gene gathered from this specimen, which is considered as the hologenetype, was deposited in Genbank with accession number MT135504.

*Type locality*—Argentina: Chubut, Biedma, Estancia El Desempeño, alongside RP 2, 33 km E RN 3 (-42.51079, -64.7471) (Fig. 1: locality 4).

*Measurements of the holotype (in mm)*—TOTL, 205; TAIL, 59.2; HFL, 27.9; EAR, 6.9; TLS, 35.38; CIL, 33.29; NL, 12.86; NW, 5.00; FL, 10.26; RW, 7.51; ZB, 21.04; IOB,6.68; BB, 15.02; BIB, 23.05; MB, 20.79; IFH, 6.41; DL, 8.54; PL, 13.95; PM4L, 2.50; TRL, 7.25. Weight, 87 g.

*Paratypes*—CFA-MA 11858 (C-5415), young female; skin, skull and partial skeleton, and CFA-MA 11868 (C-5416), young female; skin, skull and partial skeleton collected by Mabel D. Giménez, Claudio J. Bidau, Dardo A. Marti, and Martín A. Montes (field number 483 and 484, respectivelly) on 28 March 1999 at the species type locality.

*Other examined specimens*—See below in subspecies accounts.

*Morphological diagnosis*—A small to medium sized tuco-tuco of the *C. magellanicus* species group with dorsal and ventral colorations moderately differentiated; dorsum Brownish Olive to Olive or Tawny Olive; venter Pale Olive Buff with Gray colored basal hairs. Skull moderately robust, interorbital region with posteriorly divergent outer margins; premaxillo-frontal suture placed slightly to well behind from the naso-frontal suture; zygomatic arch thin to moderately robust, with slightly developed postorbital and mandibular processes of jugal and a conspicuous zygomatic depression; interparietal completely fused; incisive foramina moderately long and narrow, recessed in a common fossa of straight to slightly convex outer borders and completely separated by a thin bony septum; interpremaxillary foramen small to large; paraoccipital process fan-shaped; auditory bullae inflated, and pyrifom.

*Sperm type*—Asymmetric (Bidau, Marti & Giménez, 2002).

*Karyotype*—Specimens from Estancia El Desempeño (Fig. 1: locality 4) presented a karyotype with 2N = 38 and FN = 42, with 1 acrocentric autosomic pair, 2 submetacentric, 15 telocentric; X is acrocentric. In turn, specimens from RN 3, km 1430 (Fig. 1: locality 7) presented a karyotype with 2N = 38 and FN = 52, with 3 metacentric autosomic pairs, 4 acrocentric, 1 submetacentric, 10 telocentric; X acrocentric (Bidau, Marti & Giménez, 2002). No data is available for those populations at west-central Chubut.

*Morphological description*—Pelage dense, fine, and soft, about 14–15 mm long over back and rump; dorsum with fur ranging from Brownish Olive to Olive or Tawny Olive; individual hairs Dark Neutral Gray colored, except for the distal tips, which are lighter. Color of ventral pelage Pale Olive Buff; individual hairs Dark Gray basally, with superficial wash of Isabella. Fur of fore and hind limbs colored like dorsum and rump, except internal sides which are Isabella. Top of manus and feet covered with whitish hairs. Mystacial vibrissae surpassing the dorsal edge of the pinnae when laid back alongside of head; superciliary vibrissae sparse, extending to the base of the pinnae when laid back alongside of head. Ears sparsely covered with short, brownish hairs. Pes broad, all digits with ungueal tufts of stiff bristles, and strong claws. Tail short, darker above than below and sparsely covered by short hairs (Figs. 4B–4C). Skull moderately robust; interorbital region with posteriorly divergent outer margins; zygomatic arches nearly rounded in dorsal view; auditory bullae inflated, and pyrifom, with salient auditory tubes (Figs. 6A–6D). Nasals short, broadest anteriorly, with nearly straight lateral margins or constricted to their middle portion; premaxillary clearly visible when viewed from dorsal aspect, with the premaxillo-frontal suture slightly to well displaced behind from the naso-frontal suture (Fig. 6E). Supraorbital borders sharply defined, with slightly developed postorbitary processes on frontals. Interparietal completely fused. Temporal ridges slightly to moderately developed. Supraoccipital crest developed in adults. Zygomatic arch thin to moderately robust, with slightly to well-developed postorbital and mandibular processes of jugal and a conspicuous zygomatic depression (Fig. 6G). Incisive foramina moderately long and narrow, recessed in a common fossa of slightly convex outer borders, and completely separated by a thin bony septum; interpremaxillary foramen moderately large to absent (Fig. 6F). Palatal bridge with two major palatine foramina at about level of PM4-M1. Mesopterygoid fossae “V” shape, reaching anteriorly the middle to the anterior portion of M2. Posteropalatal pits present and very small, placed behind the M3. Alisphenoid-presphenoid bridge flat and narrow; bony roof of mesopterygoid fossa with two large sphenopalatine vacuities. Buccinator-masticatory foramen large and undivided or divided into two small foramina. Paraoccipital processes well developed and fan shaped (Fig. 6H). Upper incisors large, moderately robust, and orthodont to proodont; frontal enamel surface Orange (Fig. 1SB-E, on Data S2). Maxillary tooth rows slightly divergent posteriorly. Mandible robust, with coronoid process falciform, and strongly angled backwards; condyloid process robust; bearing a slightly developed articulation flange. Descriptive statistics for external and cranial measurements are provided on Table 4.

*Distribution*—This species has an apparently disjunct distribution, being recorded at four localities close to the Atlantic coast, between the Chubut river in the south and the Ameghino Isthmus to the north, and other two populations in west-central Chubut, south of the Chubut river (Fig. 1). Both distributional areas are about 335 km apart.

*Etymology*—This species of *Ctenomys* is named in honor of Julio R. Contreras (1933-2017), an Argentinean mammalogist and ornithologist who dedicated more than 45 years of his life to the study of the taxonomy, systematics, and biogeography of the genus *Ctenomys* (see Teta & Ríos, 2019). Contreras described more than a dozen of new species of tuco-tucos, both from Argentina and Paraguay. Together with C. Bidau (Contreras & Bidau, 1999), he authored one of the first attempts to summarize the complex evolutionary history of this genus, proposing a general hypothesis about its diversification. The species name is a patronym in the genitive singular.

*Comparisons*—*Ctenomys contrerasi* n. sp. differs from all other species of the *C. magllanicus s* pecies group by its diploid complement (see Table 2). *Ctenomys contrerasi* n. sp. is smaller than *C. fodax* (TLS ∼53 mm) and *C. magellanicus* (TLS ∼49 mm). Compared with *C. sericeus*, this tuco-tuco has proportionally narrower incisive foramina, and a different karyotype (for additional comparison, see also Figs. 3S and 4S on Data S2). It differs from *C. haigi* (2n = 50) in having a different diploid complement, a much lighter coloration and by the lack of interparietal. *Ctenomys contrerasi* n. sp. is much smaller than *C. fodax* (TLS ∼53mm) and *C. magellanicus* (TLS ∼49 mm) and has a different diploid complement from the latter. Pairwise genetic distances with other species of *Ctenomys* range from 0.8 to 4.7% (Table 1).

*Natural history*—Mostly unknown; the four coastal localities were this tuco-tuco has been collected are part of the Monte phytogeographical province, corresponding to the “Estepa de Zigofiláceas de baja cobertura (Monte Austral o Típico)”. This unit is characterized as a shrubby steppe of *Larrea divaricata*, *L. cuneifolia*, *Parkinsonia aculeata, L. ameghinoi and L. nitida*, where vegetation coverture rarely surpasses 40% (Oyarzabal et al., 2018). The other two localities are part of the Central (“Erial”) and Occidental (“Estepa arbustivo graminosa”) districts of the Patagonian phytogeographical province. Most of this area is covered by shrubby to grassy steppes, dominated by gramineous such as *Pappostipa* spp. and *Poa* spp., and low shrubs, such as *Adesmia volckmannii*, *Berberis microphylla*, *Chuquiraga* spp., *Grindelia anethifolia*, *Mulinum spinosum*, *Senecio filaginoides*, and *Nassauvia* spp. (Oyarzabal et al., 2018).

*Geographic variation*—As here defined, morphological variation in the Contreras’tuco-tuco is noticeable, reflecting consistent regional differences in size, and craniodental traits associated with differential distributions in apparently disjunct areas. However, both group of populations (i.e., coastal Chubut vs west-central Chubut) are genetically similar; haplotypes of both groups differ on average on 0.8%. Based on these differentiation levels, below we name and diagnose two subspecies of *Ctenomys contrerasi* n. sp. Additional specimens are much needed, in particular from intermediate populations, to test if, as implied in our taxonomic hypothesis, these morphotypes are the extreme of a more or less continuous gradient of variation.

### Subspecies of *Ctenomys contrerasi* n. sp.

**Table:** 

***Ctenomys contrerasi contrerasi*** n. subsp.
urn:lsid:zoobank.org:pub:59FAE1BC-4C64-4071-A58D-F0E5E477B6E1
NCR above
(Figs. 4 and 6)

*Ctenomys* [sp.]: Montes et al. (2001: 79).

*Ctenomys* [sp.]: Bidau, Marti & Giménez (2002: 64).

*Ctenomys* sp.: Nabte, Saba & Monjeau (2009: 115).

*Referred specimens .—* CFA-MA 11853 (C-05417; holotype), CFA-MA 11858 (C-5415; paratype), CFA-MA 11868 (C-5416; paratype), CNP 2, CNP 330, CNP 3601, CFA-MA 11835, CFA-MA 11848, CFA-MA 11854, CFA-MA 11855, CFA-MA 11863, CFA-MA 11864 (see File S1 for additional details).

**Figure 8:**
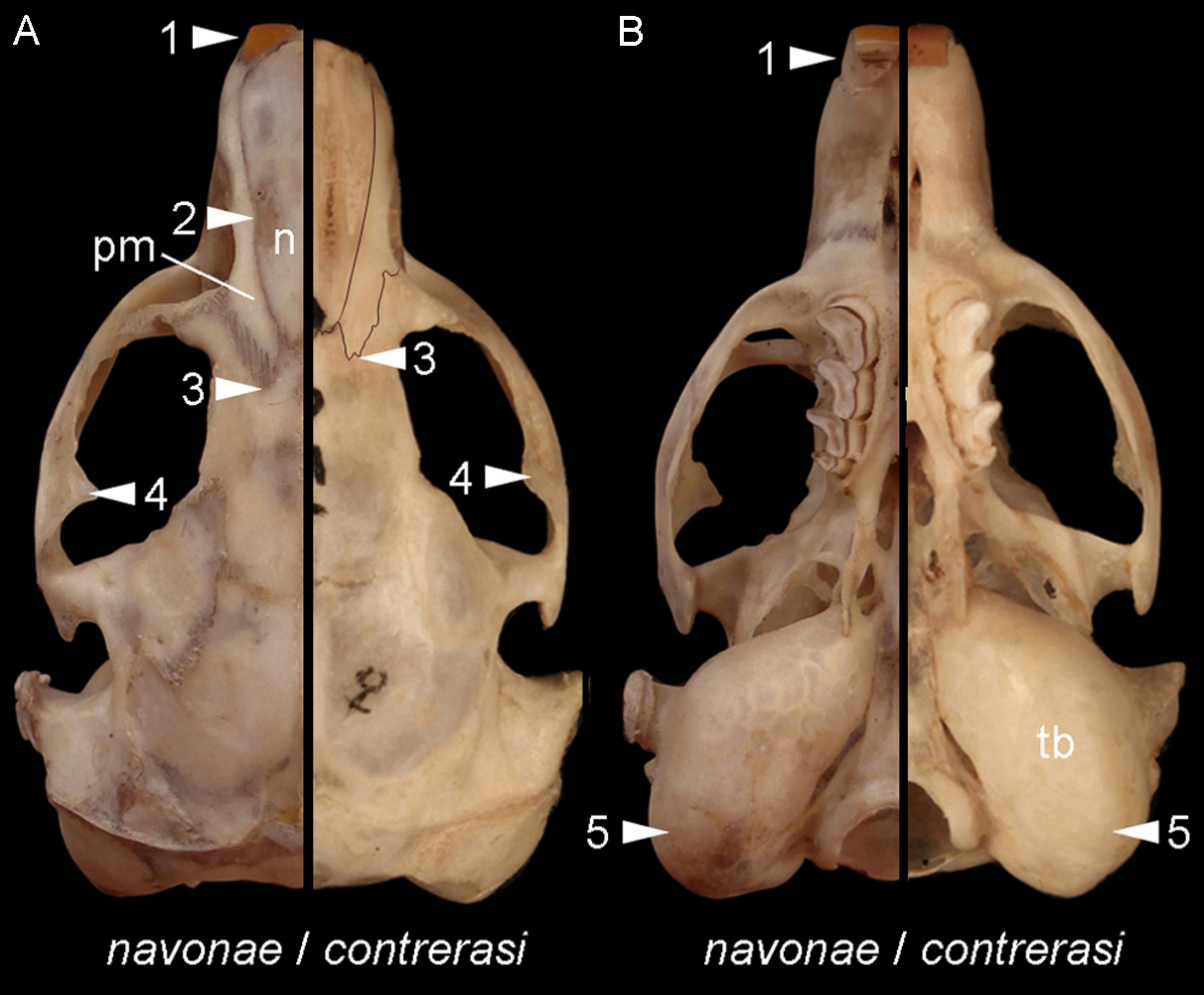
Comparison of *Ctenomys contrerasi navonae* n. subsp. and *C. contrerasi contrerasi* n. subsp. Selected differences in dorsal (A) and ventral (B) views of the cranial anatomy of *Ctenomys contrerasi navonae* n. subsp. and *C. c. contrerasi* n. subsp. The figure portrays characteristic contrasts between both taxa, including, in *C. c. navonae* n. subsp., (1) proodont incisors; (2) nasals (n) constricted towards their middle portion; (3) premaxillo-frontal suture more extended posteriorly regarding the naso-frontal suture; (4) more robust zygomatic arches; (5) less globose and proportionally smaller tympanic bullae. Individuals are not in scale to facilitate comparisons.

*Morphological diagnosis*—This subspecies is smaller and less robust than the other (see below). Overall, it has smaller and less robust zygomatic arches, with moderately expressed postorbital process of the jugal; the premaxillo-frontal suture is placed slightly behind the naso-frontal suture; the palate is shorter; the tympanic bullae are proportionally larger and more globose, and the upper incisors are nearly orthodont (see Fig. 8).

*Distribution—* Known only from four localities close to the Atlantic coast, between the Chubut river to the south and the Ameghino Isthmus to the north (Fig. 1).

**Table:** 

***Ctenomys contrerasi navonae*** n. subsp.
urn:lsid:zoobank.org:act:165495C7-44D0-4649-A29D-9AB83C32ED57
Navone’s tuco-tuco
Tuco-Tuco de Navone
WCC above
(Fig. 7)

*Ctenomys* sp. 4: Parada et al. (2011: 682).

*Ctenomys* sp. 5: Parada et al. (2011: 682).

*Holotype*—CNP 1043, adult male; skull, fluid and tissues collected on 14 December 2005 by Guillermo D’Elía (field number PNG 336). A partial (801 bp) DNA sequence of the cytochrome- b gene gathered from this specimen, which is considered as the hologenetype, was already deposited in Genbank with accession number HM777504.

*Type locality*—Argentina: Chubut, Languiñeo, Estancia Quichaura, cuadro 25 norte (-43.7017, -70.3493; 844 m).

*Measurements of the holotype (in mm)*—TOTL, 232; TAIL, 68; HFL, 32; EAR, 7; TLS, 41.91; CIL, 40.08; NL, 14.34; NW, 6.31; FL, 10.87; RW, 9.53; ZB, 24.15; IOB, 7.91; BB, 16.58; BIB, 26.07; MB, 23.70; IFH, 7.47; DL, 10.96; PL, 17.68; PM4L, 3.63; TRL, 8.74. Weight, 146 g.

*Paratype*—CNP 1437, adult female; skull, fluid and tissues collected by Daniel Udrizar Sauthier at Campo de Pichiñan, 15 km SE Cerro Condor (-43.5552 -69.0677), (field number PNG 1201) on 10 February 2006.

*Morphological diagnosis*—This subspecies is larger and more robust than *C. c. contrerasi* n. subsp. Compared with the nominotypical subspecies, it has larger and has more robust zygomatic arches, with better expressed postorbital process of the jugal; the nasals are constricted towards its middle portion; the premaxillo-frontal suture is more posteriorly placed; the palate is larger; the tympanic bullae are less globose and proportionally smaller; and the upper incisors are more proodont (see Fig. 8).

*Distribution*—Known only from two localities situated on west-central Chubut, Argentina (Fig. 1).

*Etymology*—This subspecies of *Ctenomys* is named in honor of our colleague and friend Graciela T. Navone, an Argentinean parasitologist with a large career studying small mammal endoparasites. Graciela is also a prominent and active member of the Sociedad Argentina para el Estudio de los Mamíferos (SAREM). The species name is a patronym in the genitive singular.

**Table:** 

***Ctenomys thalesi*** n. sp.
urn:lsid:zoobank.org:act:CD571C9A-330B-4565-A5E1-D8F4D37C338D
Thales’s tuco-tuco
Tuco-Tuco de Thales
SCR above
(Figs. 4 and 6)

*Ctenomys* [sp.]: Montes et al. (2001: 79).

*Ctenomys* [sp.]: Bidau, Marti & Giménez (2002: 64).

*Holotype*—CFA-MA 11849 (C-05400), adult male; skin, skull and partial skeleton collected on 23 March 1999 by Mabel D. Giménez, Claudio J. Bidau, Dardo A. Marti, and Martín A. Montes (field number 468). A partial (448 bp) DNA sequence of the cytochrome- b gene gathered from this specimen, which is considered as the hologenetype, was deposited in Genbank with accession number MT135507.

*Type locality*—Argentina: Chubut, Rawson, Establecimiento La Clara, alongside RN 1 (-43.75298, -65.44483) (Fig. 1: locality 9).

*Measurements of the holotype (in mm)*—TOTL, 215; TAIL, 65.5; HFL, 29.1; EAR, 6.8; TLS, 36.55; CIL, 35.11; NL, 11.90; NW, 5.06; FL, 11.84; RW, 7.43; ZB, 21.29; IOB, 7.00; BB, 14.96; BIB, 21.95; MB, 21.58; IFH, 6.62; DL, 8.91; PL, 15.00; PM4L, 2.72; TRL, 7.15. Weight, 96 g.

*Paratypes*—CFA-MA 11845 (C-05401), adult male; skin, skull and partial skeleton collected 23 March 1999 by Mabel D. Giménez, Claudio J. Bidau, Dardo A. Marti, and Martín A. Montes (field number 469). CFA-MA 11852 (C-05403), adult female; skin, skull and partial skeleton collected 21 March 1999 by Mabel D. Giménez, Claudio J. Bidau, Dardo A. Marti, and Martín A. Montes (field number 470). Both specimens were collected at the type locality.

*Other examined specimens*—CFA-MA 11844, CFA-MA 11846, CFA-MA 11850, CFA-MA 11862 (see File S1 for additional details).

*Morphological diagnosis*—A small-sized tuco-tuco of the *C*. *magellanicus* species group with dorsal and ventral coloration moderately differentiated; dorsum Light Brownish Olive; venter Pale Olive Buff with gray colored basal hairs. Skull moderately robust, interorbital region with posteriorly divergent outer margins; premaxillo-frontal suture placed slightly behind the naso-frontal suture; zygomatic arch thin, with slightly developed postorbital and mandibular processes of jugal and a conspicuous zygomatic depression; interparietal completely fused; incisive foramina moderately short and narrow, recessed in a common fossa of nearly convex outer borders and completely separated by a thin bony septum; interpremaxillary foramen small to absent; paraoccipital process hook-shaped; auditory bullae inflated, and pyrifom.

*Sperm type*—*Asymmetric* (Bidau, Marti & Giménez, 2002).

*Karyotype*—Specimens from La Clara (Fig. 1: locality 9) and Laguna de Indios (Fig. 1: locality 8) presented a karyotype with 2N = 28 and FN = 40, with 3 autosomic pairs acrocentric, 1 metacentric, 3 submetacentric, 6 telocentric; X acrocentric, Y metacentric (Bidau, Marti & Giménez, 2002).

*Description*—Pelage dense, fine, and soft, about 14-15 mm long over back and rump; dorsum with fur Light Brownish Olive, paler to the flanks; individual hairs Dark Neutral Gray colored, except for the distal tips, which are lighter. Some individuals have a Sepia cap on head, ca. 1.5 to 2 cm wide, running from just above nose to a line between the ears. Color of ventral pelage Pale Olive Buff; individual hairs dark gray basally, with superficial wash of Isabella. Fur of fore and hind limbs colored like dorsum and rump, except internal sides, which are Isabella. Top of manus and feet covered with whitish hairs. Mystacial vibrissae surpassing the dorsal edge of the pinnae when laid back alongside of head; superciliary vibrissae sparse, extending to the base of the pinnae when laid back alongside of head. Ears sparsely covered with short, brownish hairs. Pes broad, all digits with ungal tufts of stiff bristles, and strong claws. Tail short, darker above than below and sparsely covered by short hairs (Figs. 4E–4F). Skull moderately robust; interorbital region with posteriorly divergent outer margins; zygomatic arches broad and nearly rounded in dorsal view; auditory bullae inflated, and pyrifom, with salient auditory tubes (Figs. 6I–6L). Nasals short, broadest anteriorly, and nearly straight to slightly convex lateral margins; premaxillary clearly visible when viewed from dorsal aspect, with the premaxillo-frontal suture placed slightly behind from the naso-frontal suture (Fig. 6M). Supraorbital borders sharply defined, with slightly developed postorbitary processes on frontals. Interparietal completely fused (Fig. 6Q). Temporal ridges not developed. Supraoccipital crest moderately developed in adults. Zygomatic arch thin, with slightly developed postorbital and mandibular processes of jugal and a conspicuous zygomatic depression (Fig. 6O). Incisive foramina moderately short and narrow, recessed in a common fossa of nearly convex outer borders and completely separated by a thin bony septum; interpremaxillary foramen small to absent (Fig. 6N). Palatal bridge with two major palatine foramina at about level of M1. Mesopterygoid fossae “V” shape, reaching anteriorly the posterior portion of M2. Posteropalatal pits present and small, placed behind the M3. Alisphenoid-presphenoid bridge flat and narrow; bony roof of mesopterygoid fossa with two large sphenopalatine vacuities. Buccinator-masticatory foramen large and undivided or divided into two small foramina. Paraoccipital processes well developed and hook shaped (Fig. 6P). Upper incisors large, moderately robust, and orthodont; frontal enamel surface Orange (Fig. 1SE-F, on Data S2). Maxillary tooth rows slightly divergent posteriorly. Mandible robust (Fig. 6L), with coronoid process falciform, and strongly angled backwards; condyloid process robust; bearing a slightly developed articulation flange (Fig. 2SB, on Data S2). Descriptive statistics for external and cranial measurements are provided on Table 4.

*Distribution*—Known only from two localities on northeastern Chubut province, close to the Atlantic coast, south of Chubut river (Fig. 1).

*Etymology*—We name this species in honor of Thales Renato Ochotorena de Freitas, a Brazilian geneticist who leads a productive research program mostly centered on Brazilian species of *Ctenomys*, covering among others, aspects of taxonomy, cytogenetics, speciation, phylogeography, and conservation genetics. The species name is a patronym in the genitive singular.

*Comparisons*—Thales’s tuco-tuco differs from other closely distributed species, such as *C. bidaui* n. sp. and *C. contrerasi* n. sp. by its distinct diploid complements; in addition, *C. thalesi* n. sp. differs from the nearby distributed *C. contrerasi* n. sp. in having a proportionally shorter palate, a narrower zygomatic breadth (Fig. 3, Table 4), a proportionally narrower rostrum and nasals, a shorter upper diastema and incisive foramina, and by the shape of the paraoccipital process (Fig. 6). Compared with *C. sericeus*, its sister species, *C. thalesi* n. sp has proportionally narrower incisive foramina, a lighter coloration, and a different karyotype (for additional comparison, see also Figs. 3S and 4S on Data S2). It differs from *C. haigi* (2n = 50) in having a different diploid complement, a much lighter coloration and by the lack of interparietal. *Ctenomys thalesi* n. sp. is much smaller than *C. fodax* (TLS ∼53 mm) and *C. magellanicus* (TLS ∼49 mm) and has a different diploid complement from the latter. Pairwise genetic distances with other species of *Ctenomys* range from 2.0 to 5.0% (Table 1).

*Natural history*—Mostly unknown; Thales’s tuco-tuco, as the coastal populations of *C. contrerasi* n. sp. (*C. c. contrerasi* n. subsp.), distributes in the “Estepa de Zigofiláceas de bajacobertura (Monte Austral o Típico)”, of the Monte phytogeographical province (see the description of this vegetational unit above).

**Table:** 

***Ctenomys sericeus***Allen, 1903
Silky tuco-tuco
Tuco-Tuco sedoso
(Figs. 5S-6S, on Data S2)

*Ctenomys sericeus* Allen, 1903: 187.

*Ctenomys seriseus* Rusconi, 1928: 243; incorrect subsequent spelling.

*Ctenomys coyhaiquensis* Kelt & Gallardo, 1994:344; type locality “2 km S Chile Chico and 1 km W Chile Chico aeródromo, Provincia General Carrera, XI Región de

Aisén [= Aysén], Chile. 46°33′S, 71°46′W, 330 m”.

*Holotype*—USNM 84191, adult male; skin and skull collected on 5 February 1897 by O. A. Peterson.

*Type locality*—Argentina: Santa Cruz “Cordilleras, upper Rio Chico de Santa Cruz, Patagonia”; restricted to “confluencia de los ríos Belgrano y Chico (∼48.26°S, 71.20°W, departamento Río Chico, Santa Cruz, Argentina” by Pardiñas (2013).

*Morphological diagnosis*—pelage short, soft, and glossy (Fig. 5S, on Data S2); general color above Olive Brown to Sepia strongly varied with Black, the hairs being Dark Gray for the basal three fourths, then banded narrowly with pale Yellowish Brown, and tipped with Black; top of nose and top of head like median dorsal region, which is darker than the sides, sometimes forming a dark median dorsal band extending from the nose to the base of the tail; flanks lighter than dorsum and venter Isabella; ears very small, blackish; upper surface of feet grayish to yellowish; tail Tawny Olive, with a median dusky stripe along the apical half of the upper surface. Skull moderately robust (Fig. 6S, on Data S2), interorbital region with posteriorly divergent outer margins; premaxillo-frontal suture placed behind from the naso-frontal suture; zygomatic arches robust, with conspicuously and moderately developed postorbital and mandibular processes of jugal, respectively, and a well-marked zygomatic depression; interparietal absent to very small; incisive foramina moderately short and broad, recessed in a common fossa of convex outer borders and completely separated by a thin bony septum; interpremaxillary foramen large to inconspicuous; paraoccipital hook-shaped; auditory bullae inflated, and pyrifom.

*Sperm type—* Asymetric (Bidau, 2015)

*Karyotype*—2n = 28-30; FN = 44-46 (Kelt & Gallardo, 1994; Bidau, 2015).

*Distribution*—*C. sericeus* occurs in open shrubby to herbaceous steppes from southwestern Chubut (Argentina) in the north to the northern margin of the Santa Cruz river (Santa Cruz, Argentina) in the south, and adjacent open areas of Aysen, Chile (Fig. 1).

*Morphological comparisons*—See the comments on previous accounts and Figs. 3S and 4S on Data S2.

*Remarks—* Based on its geographical provenance, the gene tree topology, and the description provided by Vincón (2004), we considered that the sample identified by Parada et al. (2011) as “*C. fodax*” and referred by Teta, D’Elía & Opazo (2020) as *C.* sp. 1 (and as *C.* aff *C. sericeus* above) belongs to *C. sericeus*.

*Natural history*—Mostly unknown; known localities in Argentina are mostly included within the Central (“Erial”) and Occidental (“Estepa arbustivo graminosa”) districts of the Patagonian phytogeographical province. This area is covered by shrubby to grassy steppes, dominated by grasses such as *Pappostipa* spp. and *Poa* spp., and low shrubs, such as *Adesmia volckmannii*, *Berberis microphylla*, *Chuquiraga* spp., *Grindelia anethifolia*, *Mulinum spinosum*, *Senecio filaginoides*, and *Nassauvia* spp. (Oyarzabal et al., 2018). Similarly, Chilean localities were *C. sericeus* has been collected present well-drained rocky and sandy soils with a sparse vegetal cover of shrub and herbs (Kelt & Gallardo, 1994).

## Discussion

Almost 90 years ago, the distinguish British mammalogist Olfield Thomas (1929) wrote: “…the whole of the tuco-tucos obtained by Sr. Budin south of the Rio Negro appear to belong to but a single species, for which Dr. Allen’s name of *C. sericeus* is available”. Among the animals studied by Thomas (1929) there were specimens from eastern Río Negro, eastern Chubut, and northeastern Santa Cruz, including some caught close to some localities sampled in this study. Although it is possible that most of the populations studied by Thomas belong to the lineage of *C. sericeus*, at the light of the new species uncovered in this study, their taxonomic status should be further assessed. This task should not be only based on a qualitative morphologic approach; as shown here, an integrative approach is needed to adequately characterize the specific diversity of *Ctenomys* (e.g., Freitas et al., 2012; Gardner, Salazar Bravo & Cook, 2014).

The integration of karyological, morphological, and genetic data conducted in this study, clearly indicates that the species diversity of Patagonian *Ctenomys* was not reflected in the previous taxonomic scheme. As such, in this study we named and described three new species, one including two subspecies, of tuco-tucos, at the time that formally posed the synonymy of a Chilean form under a species widely distributed in the Argentinean Patagonia.

The three new species of tuco-tucos described here, *C. bidaui* n. sp., *C. contrerasi* n. sp., and *C. thalesi* n. sp., are clearly distinguished from each other as well as from the other Patagonian species. Each new species represents a distinct and divergent lineage in the cytb genealogy. In addition, we recognize two subspecies under *C. contrerasi* n. sp., *C. c. contrerasi* n. subsp., and *C. c. navonae*, n. subsp. Both forms are morphologically distinctive (Figs. 3 and 8; Table 2), although genetically they diverge from each other only by 0.8%. This certainly is a low value, although there are pair of species of *Ctenomys* that show even lower values; for example, *C. “yolandae”* and *C. bergi* share the same haplotype of the cytb (Mascheretti et al., 2000), while *C. scagliai* and *C. saltarius* diverge at this gene by 0.6% (Parada et al., 2011). As such, both forms could be ranked at the species level. However, we prefer to advance a more conservative taxonomic scenario considering incomplete knowledge about the morphological variation within these lineages due to the small samples sizes here studied, and the fact that there is a large unsampled area between the known distributions of both forms that preclude us from discard a scenario in which both morphotypes are part of a large cline of morphological variation. In addition, we note that, together with the observed low divergence value, in the gene trees both forms have low support. Finally, the lack of karyotypic data and other morphological traits (e.g., type of sperm) for *C. c. navonae* n. subsp. hampers comparing it in these features with the *C. c. contrerasi*. Therefore, here we recognize both forms as distinct subspecies, awaiting the availability of additional data to further assess their distinction. With the exception noted, the other three nominal forms show a distinct karyotype, being different among them and with previous known species of the *C. magellanicus* species group. Karyotipically the most similar species are *C. thalesi* n. sp. and *C. sericeus*; the former shows 2n = 28, a number presented in the variation of *C. sericeus* (2n =28 − 30), but both species differ in their fundamental numbers (40 vs. 44-46, respectively) and are phenotypically distinct (Table 2). Finally, the four new species differ morphological from each other as well as from the other species of the *C. magellanicus* species group.

Based both on molecular and morphological evidence, we placed *C. coyhaiquensis* under the synonymy of *C. sericeus*. *Ctenomys coyhaiquensis* has a 2n = 28; FN = 44 (Kelt & Gallardo, 1994), a complement that is included within the known range of variation of *C. sericeus* (2n = 28-30, FN 44-46; Montes et al., 2001; Bidau, 2015). In addition, both nominal forms have sperms of the type simple asymmetric. Haplotypes of both forms differ on average by 0.7%; in addition, haplotypes of each nominal form do not form well supported clades (Fig. 2). It is important to note than when described (Kelt & Gallardo, 1994), *C. coyhaiquensis* was compared in detail with the closely distributed *C. colburni* (= *C. magellanicus*; see Teta, D’Elía & Opazo, 2020) but not with *C. sericeus*. Given our taxonomic scheme (see also Teta, D’Elía & Opazo, 2020), three species of *Ctenomys*, *C. fodax*, *C. magellanicus*, and *C. sericeus*, are currently known from Chilean Patagonia.

Although is not the focus of this contribution, we remark the fact that haplotypes of *C. haigi* do not form a monophyletic group (see also Parada et al., 2011). As is currently delimited, this species encompasses two main lineages, morphologically similar, that would correspond to two different species (Fig. 2). We choose to label these clades as *C. haigi* s.s., for the lineage represented by the haplotype of a specimen collected at the type locality of the species, and *C.* cf. *C. lentulus*, for the most widely distributed lineage, which included within its range the type locality of *lentulus* Thomas (the closest studied sample [Fig. 1: locality 4] to the type locality is from ca. 26 km W). Again, we advocate for the need of additional evidence, including new sequences and the inspection of the holotypes of both nominal forms, to further advance this question. This issue constitutes an example of the multiple aspects that remain so far unsolved in the taxonomy of *Ctenomys*.

From north to south, in Argentinean and Chilean Patagonia, the diversity of the genus *Ctenomys* decreases from seven recognized species between 42°−46°S (six corresponding to the *magellanicus* group plus *C. sociabilis*), to two between 46°−50°S (*C. magellanicus* and *C. sericeus*), and only one south of 50°S (*C. magellanicus*) (cf. Bidau, 2015). Two of the four taxa described in this contribution, *C. bidaui* n. sp. and *C. c. contrerasi* n. subsp., occur north of the Chubut River, while the other two, *C. c. navonae* n. subsp. and *C. thalesi* n. sp., are present south of this watercourse. In addition, all known records of *C. sericeus* lay north of the Santa Cruz River and, most of them, south of the Deseado River. Other populations referred to *C. sericeus*, such as those on southeastern Chubut and eastern Río Negro (Thomas, 1929), need to be adequately studied, since our morphological inspection of individuals from different localities (e.g., Pico Salamanca, Valcheta) suggests that they could correspond to other innominate forms. As with other morphologically similar species, additional evidence, such as DNA sequences and karyotypes, are much needed to evaluate with more certainty the taxonomic status of these populations.

Our phylogenetics analysis based on mitochondrial DNA sequences of all currently known species *C*. *magellanicus* species group shows that they fall into three main lineages (Fig. 2). One lineage is composed exclusively by *C. bidaui* n. sp. and is therefore restricted to Península de Valdés, Chubut, Argentina. The second lineage, being widely distributed in most of southern Patagonia and Tierra del Fuego, includes *C. magellanicus* and, much probably, *C. fodax* (see Teta, D’Elía & Opazo, 2020) for which no sequence is available. The third and last main lineage, distributed north of the Santa Cruz River, to at least the provinces of Neuquén and Río Negro, is the most species rich of the three, being conformed by *C. haigi* s.l., *C. contrerasi* n. sp. (including *C. c. contrerasi* n. subsp., and *C. c. navonae* n. subsp.), *C. sericeus* (including *C. coyhaiquensis*, see discussion below), and *C. thalesi* n. sp. (Fig. 1).

A detailed study of the genus *Ctenomys* across larger areas of northern Patagonia is still much needed, not only to determine the real species diversity of Patagonian *Ctenomys*, but also to accurately delimit the geographic distribution of the already known species. For example, most references for northern Patagonia were made using an open taxonomy, a situation that hampers studies in other areas (e.g., Nabte, Saba & Monjeau, 2009; Udrizar Sauthier & Pardiñas, 2014). In addition, there are some old references that appear to be erroneous at the light of current knowledge of this genus (e.g., *C. magellanicus* for Punta Tombo, eastern Chubut Thomas, 1898). After pending issues on the alpha diversity of Patagonian *Ctenomys* be solved, the distinction and contents of these main lineages should be further evaluated; in turn, the resulting phylogenetic hypothesis would be the basis to advance a biogeographic scenario of the historical biogeography of the genus in Patagonia.

Finally, it is important to note that, as currently defined, the four new species described here have small geographical ranges, which constitute an indirect measure of their conservation status (Bidau, Lessa & Ojeda, 2012). In fact, it is possible that some of the new species described here could be considered as endangered or vulnerable, due to the small distributional areas that they occupy within a region highly impacted by human activities (e.g., Aagessen, 2000), where large scale extractivism is increasingly being developed (e.g., mega-mining; see Jerez, 2017; Weinstock, 2017). Additional surveys are much needed, in order to detect new populations of these taxa to accurately define their distributions and conservation statuses.

## Conclusions

The integrative analyses of morphological, molecular, and karyotipic data of Patagonian specimens of *Ctenomys* allowed as to describe three new species endemics to the open areas of northern Patagonia. The three new species belong to the *C. magellanicus* species group. In addition, we consider the geographically restricted *C. coyhaiquensis* (Kelt & Gallardo, 1994) as a junior synonym of the widespread *C. sericeus* (Allen, 1903). Our results also shown that as currently understood, *C. haigi* is likely a composite of two lineages of species level; tentatively, we refer to then as *C. haigi* s.s. and *C.* cf. *C. lentulus*. Our findings, together with the fact that large Patagonian areas still remain unstudied, suggest that the diversity of Patagonian species of *Ctenomys* is only partially understood. Therefore, to fill in this gap of knowledge, it is needed to carry out additional integrative taxonomic studies, based on the field collection of additional specimens.

##  Supplemental Information

10.7717/peerj.9259/supp-1 File S1 List of analyzed specimens of *Ctenomys*

10.7717/peerj.9259/supp-2 File S2 Individual measurements of specimens of *Ctenomys* used in the morphological analyses

10.7717/peerj.9259/supp-3 Data S1 Supplemental Tables

10.7717/peerj.9259/supp-4 Data S2 Supplemental Figures

10.7717/peerj.9259/supp-5 Data S3 New DNA Sequences of *Ctenomys* Genbank accession numbers: MT135501 to MT135507

## Additional Information and Declarations

### DNA Deposition

The following information was supplied regarding the deposition of DNA sequences: The Cytochrome-b sequences are available at GenBank: MT135501 to MT135507.

### Data Availability

The following information was supplied regarding data availability: The list of specimens of *Ctenomys* and the details of the collection where those specimens are deposited are available in File S1. The raw measurements of specimens of *Ctenomys* assessed in this study are available in File S2.

### New Species Registration

The following information was supplied regarding the registration of a newly described species: Publication LSID: urn:lsid:zoobank.org:pub:59FAE1BC-4C64-4071-A58D-F0E5E477B6E1 *Ctenomys bidaui* n. sp. LSID: urn:lsid:zoobank.org:act:B90C9672-525E-4E00-BA69-0EE58F4159E8 *Ctenomys contrerasi* n. sp. LSID: urn:lsid:zoobank.org:act:14D8E298-5175-4688-A1EC-105F81163132 * Ctenomys contrerasi contrerasi* n. subsp. LSID: urn:lsid:zoobank.org:act:3CDD3C62-1C8C-4ED1-882B-5269E336A823 *Ctenomys contrerasi navonae* n. subsp. LSID: urn:lsid:zoobank.org:act:165495C7-44D0-4649-A29D-9AB83C32ED57 *Ctenomys thalesi* n. sp. LSID: urn:lsid:zoobank.org:act:CD571C9A-330B-4565-A5E1-D8F4D37C338D
