# Supplementary material for: Uncovering the species diversity of subterranean rodents at the end of the World: three new species of Patagonian tuco-tucos (Rodentia, Hystricomorpha, Ctenomys)
Source: PeerJ. 2020 May 29;8:e9259. doi: 10.7717/peerj.9259 (PMC7263298; doi:10.7717/peerj.9259)
Supplement: File S1 [file peerj-08-9259-s001.doc]

**Supplemental Information S1:** List of analyzed specimens.

List of specimens of *Ctenomys* included in genetic (Genbank accession numbers are included between parenthesis; sequences gathered here are indicated with an * next to Genbank accession numbers) and morphologic (indicated with an ^m^) based analyses. Species allocation follows the taxonomic scheme here proposed (see text). For each specimen of the *Ctenomys magellanicus* species group we provide locality information, catalog number, and Genbank accession number. Acronyms are as follow: CNP, Centro Nacional Patagónico (Chubut, Argentina); FMNH, Field Museum of Natural History (Chicago, U.S.); CFA-MA, Fundación de Historia Natural “Félix de Azara” (Buenos Aires, Argentina); MACN-Ma, Museo Argentino de Ciencias Naturales “Bernardino Rivadavia” (Buenos Aires, Argentina); UACH, Universidad Austral de Chile (Valdivia, Chile); USNM, U. S. National Museum of Natural History, Smithsonian Institution (Washington DC, U.S.). Numbers between brackets correspond to those depicted in Fig. 1.

***Ctenomys bidaui*** n. sp*.—*Argentina: Provincia de Chubut, Departamento Biedma, 1 km W Punta Delgada, sobre RP 2 (CFA-MA 12281 MT135502, CFA-MA 12282 MT135503 [12]), Departamento Biedma, Golfo San José al E (MACN-Ma 16395 [10]), Departamento Biedma, Puerto Pirámides (CFA-MA 11857^m^/ MT135501, CFA-MA 11865^m^, CFA-MA 11867^m^ [holotype] [11]).

***Ctenomys contrerasi contrerasi*** n. subsp*.—*Argentina, Provincia de Chubut, Departamento Biedma, RP 2, 33 km E RN 3, Estancia El Desempeño (CFA-MA 11853^m^/MT135504 [holotype], CFA-MA 11858^m^, CFA-MA 11868^m^/MT135505 [13]), Departamento Biedma, RN 3, km 1430 (CFA-MA 11835^m^, CFA-MA 11848^m^, CFA-MA 11854^m^, CFA-MA 11855^m^, CFA-MA 11863^m^, CFA-MA 11864^m^ [16]), Departamento Biedma, RP 4 (CNP 2^m^, CNP 330^m^ [14]), Departamento Biedma, Punta Este (CNP 3601^m^ [15]).

***Ctenomys contrerasi navonae*** n. subsp*.—*Argentina: Provincia de Chubut, Departamento Languiñeo, Estancia Quichaura (CNP 1043^m^/HM777504 [holotype] [18]), Departamento Paso de Indios, Pichiñan (CNP 1437^m^/HM777503 [17]).

***Ctenomys haigi*** s.s*.—*Argentina: Provincia del Chubut, Departamento Cushamen, El Maitén, 42°3’S 71°10’W (SV62/HM777476; topotype [1]),

***Ctenomys* cf.** ***C.*** ***lentulus****.—*Argentina: Provincia del Chubut, Departamento Cushamen, Laguna Nahuelquir, Estancia El Maitén (MNT018/KU659607, SM01/KU659602, SM02/KU659603, SM03/KU659604, SM04/KU659605, SM05_KU659606 [9]), Departamento Telsen, Talagapa (CNP 1269/HM777505 [8]); Provincia del Neuquén, Departamento Los Lagos, Cueva Traful (HA2C222/GU433041, HA2AC61/GU433042, HAC201/GU433043, HA3C83/GU433044, HA1AC266/GU433045, HA4C62/GU433046 [2]); Provincia de Rio Negro, Departamento Bariloche, Bariloche (MHNG1276071/KU659608 [6]), Departamento Bariloche, near Hipodromo, 13 km WNW Bariloche (MVZ 166421/AF007063 [5]), Departamento Pilcaniyeu, 13.5 km E Estación Perito Moreno (MVZ 184878/AF422920 [4]), Departamento Pilcaniyeu, Estancia San Ramón (H047_KY013599 [3]); Departamento Valcheta, Cerro Corona (CNP 3610/HM777506 [7]).

***Ctenomys magellanicus****.—*Argentina: Provincia de Santa Cruz, Departamento Lago Buenos Aires, Estancia La Cantera (UACH 4232/MN176514*, UACH4233/MN176515* [30]), Departamento Lago Buenos Aires, Río Ecker, 500 m aguas abajo casco Ea. Casa de Piedra (CNP 3613/HM777474 [31]), Río Chico, Estancia El Puma (CFA-MA 11332/MN176516*, CFA-MA11346/MN176517* [33]). Provincia de Tierra del Fuego, Departamento de de Río Grande, no locality neither voucher specimen especified (DQ333326, DQ333327), Departamento de de Río Grande, Estancia Sara (CNP 3594/HM777479 [35]); Chile: Provincia de Capitán Prat, Parque Patagonia (UACH 8087/MN176518*, UACH 8088/MN176519* [32]); Provincia de Última Esperanza, Parque Nacional Torres del Paine, Laguna Amarga (UACH 8089/MN176520*, UACH 8090/MN176521*, UACH 8091/MN176522*, UACH 8092/MN176523*, UACH 8093/MN176524*, UACH 8094/MN176525*, UACH 8095/MN176526*, UACH 8096/MN176527* [34]); Provincia de Tierra del Fuego, Tres Arroyos (I308/AF370690 [36]).

***Ctenomys sericeus***.—Argentina: Provincia de Chubut, Departamento Río Senguer, Lago Blanco (SV52/HM777475); Provincia de Santa Cruz, Departamento Deseado, 1 km Estancia la Paloma, sobre RP 37 (CNP 3604^m^/HM777501 [24]), Departamento Deseado, Cerro Condor (CNP 1416^m^ [23]), Departamento Deseado, Cerro del Paso (CNP 3605^m^/HM777502 [25]), Departamento Corpen Aike, La Porteña, Río Lista (SV45/HM777496 [27]), Departamento Río Chico, Upper río Chico (USNM 84187^m^, USNM 84189^m^ [holotype], USNM 84193^m^, USNM 84194^m^, USNM 132277^m^, USNM 147922^m^, USNM 257437^m^ [26], Departamento Río Chico, Estancia Las Tunas, Lago Cardiel (PPA 776^m^ [28]), Departamento Río Chico, Cerro Ventana (CNP 803^m^, CNP 3590^m^, CNP 3602^m^, CNP 3609^m^, CNP 3612^m^, CNP 3614^m^, CNP 3615^m^/HM777500, CNP 3616^m^ [29]). Chile: Región de Aysen, Provincia General Carrera, Provincia General Carrera, Chile Chico, 2 km SEE (FMNH 134264/AF119112, FMNH 134296/AF119113, FMNH 134300/ AF071753, UACH 3642^m^, UACH 3644^m^, UACH 3646^m^, UACH 3647^m^, UACH 3648^m^, UACH 3649^m^, UACH 3650^m^, UACH 3651^m^, UACH 3652^m^, UACH 3653^m^, UACH 3654^m^, UACH 4434^m^, UACH 4435^m^, UACH 4437^m^, UACH 4438^m^, UACH 4439^m^, FMNH 134270^m^, FMNH 134271^m^, FMNH 134274^m^, FMNH 134276^m^, FMNH 134277^m^, FMNH 134278^m^, FMNH 134279^m^ [holotype of *Ctenomys coyhaiquensis*], FMNH 134295^m^, FMNH 46135^m^ [22]), Fundo Los Flamencos (UACH 4429^m^, UACH 4430^m^, UACH 4431^m^, UACH 4432^m^, UACH 4433^m^ [21]).

***Ctenomys thalesi*** n. sp.—Argentina: Provincia de Chubut, Departamento Rawson, Establecimiento La Clara, sobre RP 1 (CFA-MA 11845^m^, CFA-MA 11849^m^/MT135507 [holotype], CFA-MA 11852^m^ [20]), Departamento Rawson, Estancia Laguna de los Indios, sobre RN 1 (CFA-MA 11844^m^, CFA-MA 11846^m^, CFA-MA 11847/MT135506, CFA-MA 11850^m^, CFA-MA 11862^m^ [19]).

**Outgroup***.—Ctenomys torquatus* (CA743/AF119111); *Ctenomys tucumanus*, (C04670/HM777499); *Ctenomys boliviensis* (NK15726/AF007038); *Ctenomys sociabilis* (EAL545/HM777495).
