# Supplementary material for: Uncovering the species diversity of subterranean rodents at the end of the World: three new species of Patagonian tuco-tucos (Rodentia, Hystricomorpha, Ctenomys)
Source: PeerJ. 2020 May 29;8:e9259. doi: 10.7717/peerj.9259 (PMC7263298; doi:10.7717/peerj.9259)
Supplement: Data S1 [file peerj-08-9259-s003.doc]

**Data S1**: Supplementary tables.

**Table 1S.** Summary statistics (mean. SD. range) of cranial measurements (in mm) of adult samples (n) of *Ctenomys coyhaiquensis* and *C. sericeus.* See Materials and Methods for explanation of the abbreviations.

|  | ***C. coyhaiquensis*** | | | | | ***C. sericeus*** | | | | |
| --- | --- | --- | --- | --- | --- | --- | --- | --- | --- | --- |
|  | **n** | **Mean** | **SD** | **Min.** | **Max.** | **n** | **Mean** | **SD** | **Min.** | **Max.** |
| **TLS** | 31 | 39.55 | 2.04 | 34.73 | 43.41 | 18 | 37.90 | 2.09 | 34.84 | 43.83 |
| **CIL** | 31 | 37.55 | 2.15 | 32.94 | 41.43 | 18 | 35.77 | 2.09 | 32.42 | 41.04 |
| **NL** | 31 | 13.76 | 1.02 | 10.66 | 15.58 | 18 | 12.96 | 0.79 | 11.68 | 14.31 |
| **NW** | 31 | 5.53 | 0.50 | 4.81 | 7.13 | 18 | 5.46 | 0.45 | 4.55 | 6.65 |
| **FL** | 31 | 12.13 | 0.66 | 10.64 | 13.44 | 18 | 12.01 | 0.63 | 10.78 | 13.26 |
| **RW** | 31 | 8.21 | 0.79 | 5.04 | 9.73 | 18 | 7.79 | 0.68 | 6.76 | 9.55 |
| **ZB** | 31 | 22.72 | 1.12 | 20.48 | 25.16 | 18 | 21.91 | 1.30 | 20.00 | 25.75 |
| **IOB** | 31 | 7.23 | 0.33 | 6.54 | 8.08 | 18 | 7.01 | 0.53 | 6.14 | 8.53 |
| **BB** | 31 | 15.99 | 0.39 | 15.19 | 16.80 | 18 | 15.57 | 0.71 | 14.23 | 16.83 |
| **BIB** | 31 | 23.37 | 1.03 | 21.40 | 25.58 | 18 | 23.54 | 1.36 | 21.21 | 26.17 |
| **MB** | 31 | 22.47 | 0.94 | 20.25 | 24.56 | 18 | 22.17 | 1.07 | 20.29 | 24.88 |
| **IFH** | 31 | 7.33 | 0.48 | 6.21 | 8.65 | 18 | 6.94 | 0.53 | 6.08 | 8.11 |
| **DL** | 31 | 10.21 | 1.02 | 7.75 | 11.92 | 18 | 9.71 | 0.83 | 8.49 | 12.17 |
| **PL** | 31 | 16.66 | 1.26 | 12.96 | 18.90 | 18 | 15.79 | 1.22 | 14.14 | 19.12 |
| **PM4L** | 31 | 3.06 | 0.15 | 2.76 | 3.37 | 18 | 3.09 | 0.24 | 2.70 | 3.64 |
| **TRL** | 31 | 7.90 | 0.49 | 6.74 | 8.88 | 18 | 7.63 | 0.52 | 6.69 | 8.55 |

**Table 2S**. Results of principal components analyses (first and second columns) and discriminant function analysis (third and fourth columns) performed on five geographical groups of adult specimens of *Ctenomys coyhaiquensis* and *C. sericeus* (n = 47). See Materials and Methods for explanation of abbreviations.

|  | **PC 1** | **PC 2** | **CV1** | **CV2** |
| --- | --- | --- | --- | --- |
| **TLS** | 0.2480 | 0.0886 | -190.77 | -40.465 |
| **CIL** | 0.2656 | 0.1176 | 30.761 | -80.048 |
| **NL** | 0.2962 | 0.1694 | -1.9677 | -12.177 |
| **NW** | 0.2641 | -0.8135 | 21.829 | 7.5786 |
| **FL** | 0.0742 | 0.2122 | -3.9529 | 1.7836 |
| **RW** | 0.2920 | 0.0867 | -18.537 | -21.169 |
| **ZB** | 0.2423 | 0.0342 | -25.188 | 1.8507 |
| **IOC** | 0.1904 | -0.1922 | 5.221 | 14.592 |
| **BB** | 0.1086 | -0.0380 | 6.6689 | -43.367 |
| **BIB** | 0.1785 | -0.0441 | -62.668 | 69.579 |
| **MB** | 0.1805 | -0.0462 | 90.677 | 40.241 |
| **IFH** | 0.2801 | -0.0290 | 51.514 | -18.463 |
| **DL** | 0.4172 | -0.0380 | 28.357 | 21.09 |
| **PM4L** | 0.1469 | 0.1585 | 1.2345 | 23.557 |
| **PL** | 0.3466 | 0.0783 | 46.108 | 25.91 |
| **TRL** | 0.2328 | 0.3976 | -27.997 | 5.6386 |
| **Eigenvalue** | 0.008 | 0.001 | 5.2859 | 3.3490 |
| **% variance** | 66.79 | 79.25 | 50.11 | 31.75 |
