# Supplementary material for: Uncovering the species diversity of subterranean rodents at the end of the World: three new species of Patagonian tuco-tucos (Rodentia, Hystricomorpha, Ctenomys)
Source: PeerJ. 2020 May 29;8:e9259. doi: 10.7717/peerj.9259 (PMC7263298; doi:10.7717/peerj.9259)
Supplement: Data S2 [file peerj-08-9259-s004.doc]

**Data S2:** Supplementary figures.


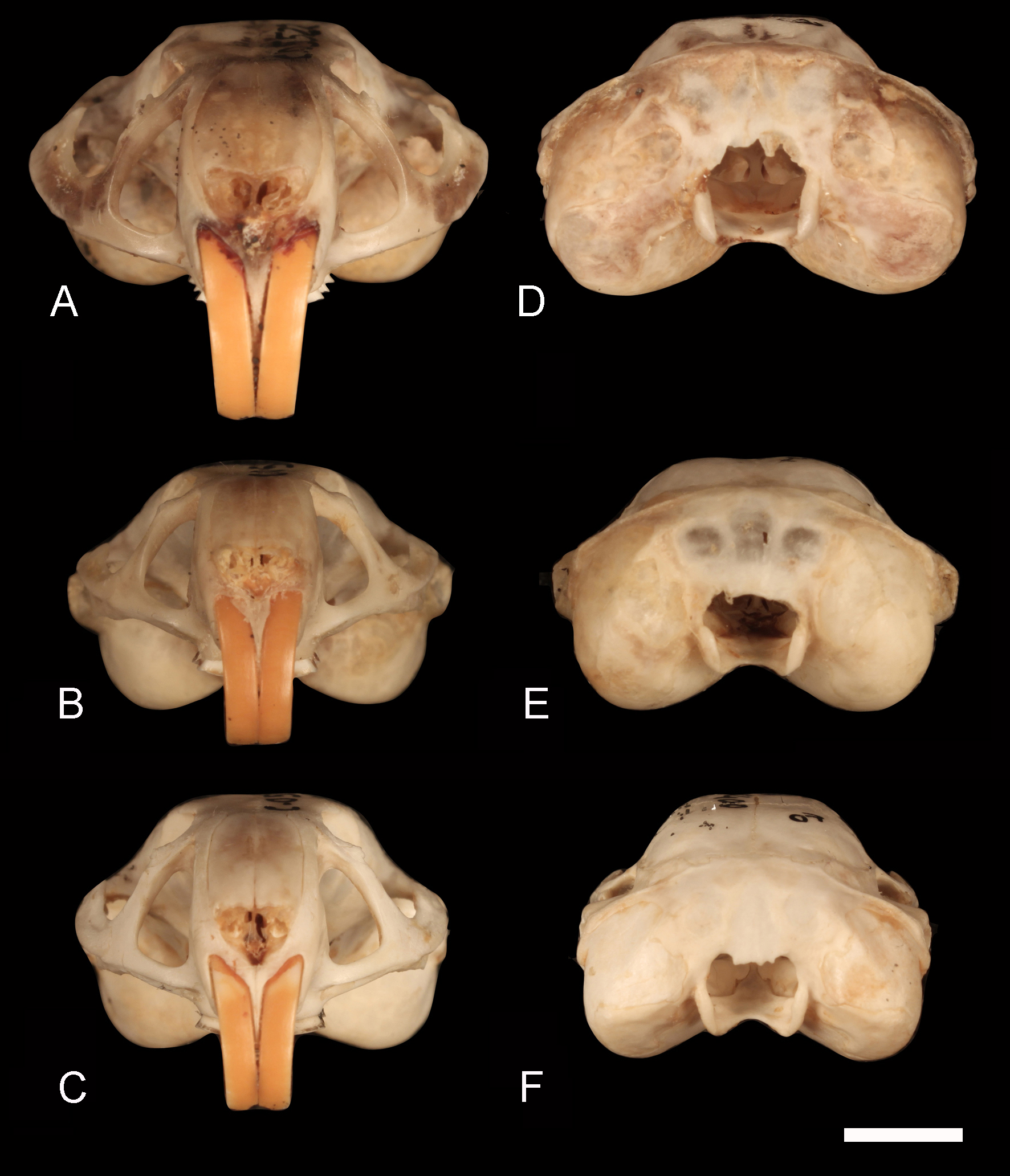


**Figure 1S.** In face (A-C) and occipital (D-F) views of the skulls of the holotypes of *C. bidaui* n. sp. (A, D; CFA 11867), *C. contrerasi* n. sp. (B, E; CFA 11853), and *C. thalesi* n. sp. (C, F; CFA 11849). Scale = 5 mm.


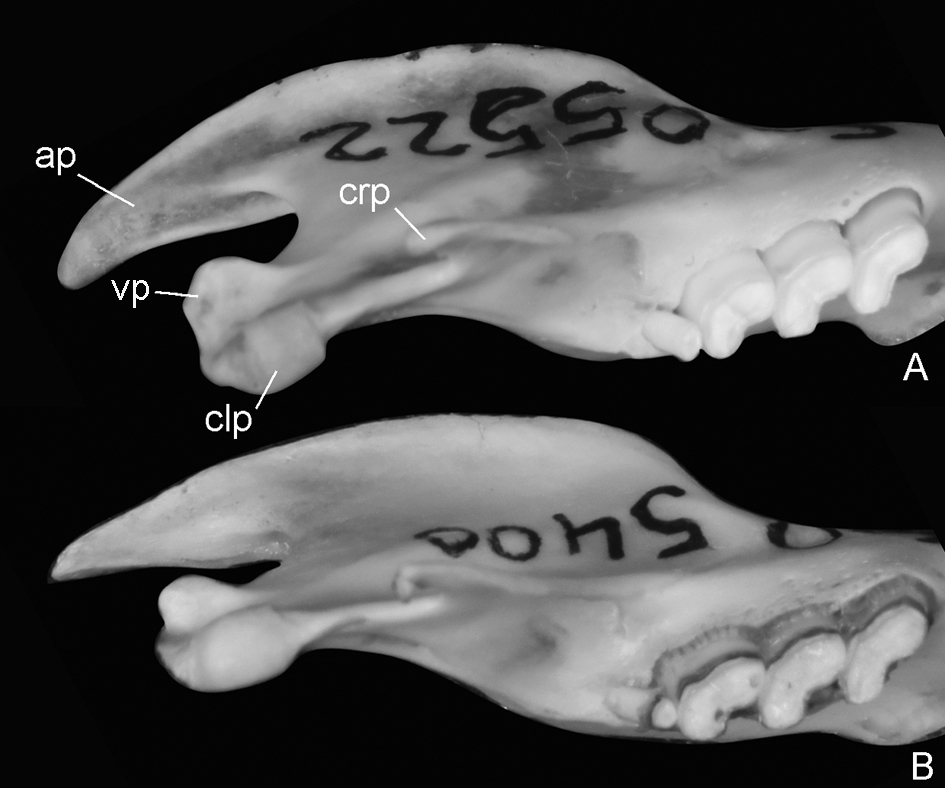


**Figure 2S.** Dorsal view of the posterior portion of the mandibles of the holotypes of *Ctenomys bidaui* n. sp. (A; CFA 11867), and *C. thalesi* n. sp. (B; CFA 11849). Abbreviations: af, articulation flange; crp, coronoid process; clp, condyloid process; va, ventrolateral apophysis of the postcondyloid process. Images are not in scale to facilitate comparisons.


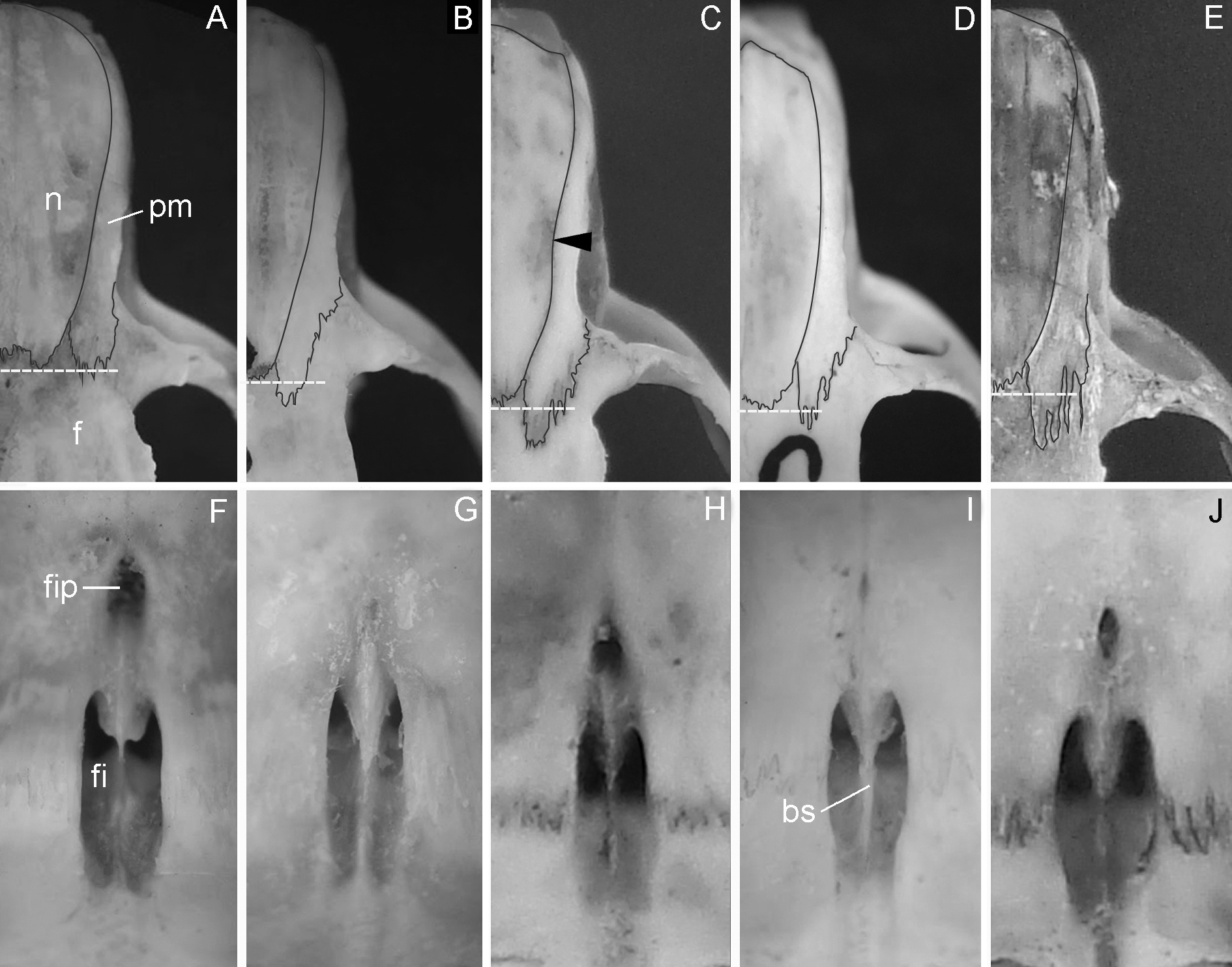


**Fig. 3S.** Selected cranial traits of *Ctenomys* (A, F, holotype of *C*. *bidaui* n. sp. [CFA 11867]; B, G, holotype of *C*. *c. contrerasi* n. sp. [CFA 11853]; C, H, holotype of *C*. *contrerasi navonae* n. sp. [CNP 1043]; D, I, *C*. *thalesi* n. sp. [CFA 11849]; and E, J, *C. sericeus* [CNP 3612]): A-E) nasals (n) and premaxillae (pm); the broken white line depict the posterior border of nasals, the black arrow in D show the constriction of the nasals toward its middle portion; F-J) incisive (fi) and interpremaxillary (fip) foramina. Figures in A-E are not in scale to facilitate comparisons; figures in F-J are scaled to the same diastema length. Other abbreviations: bs, bony septum.


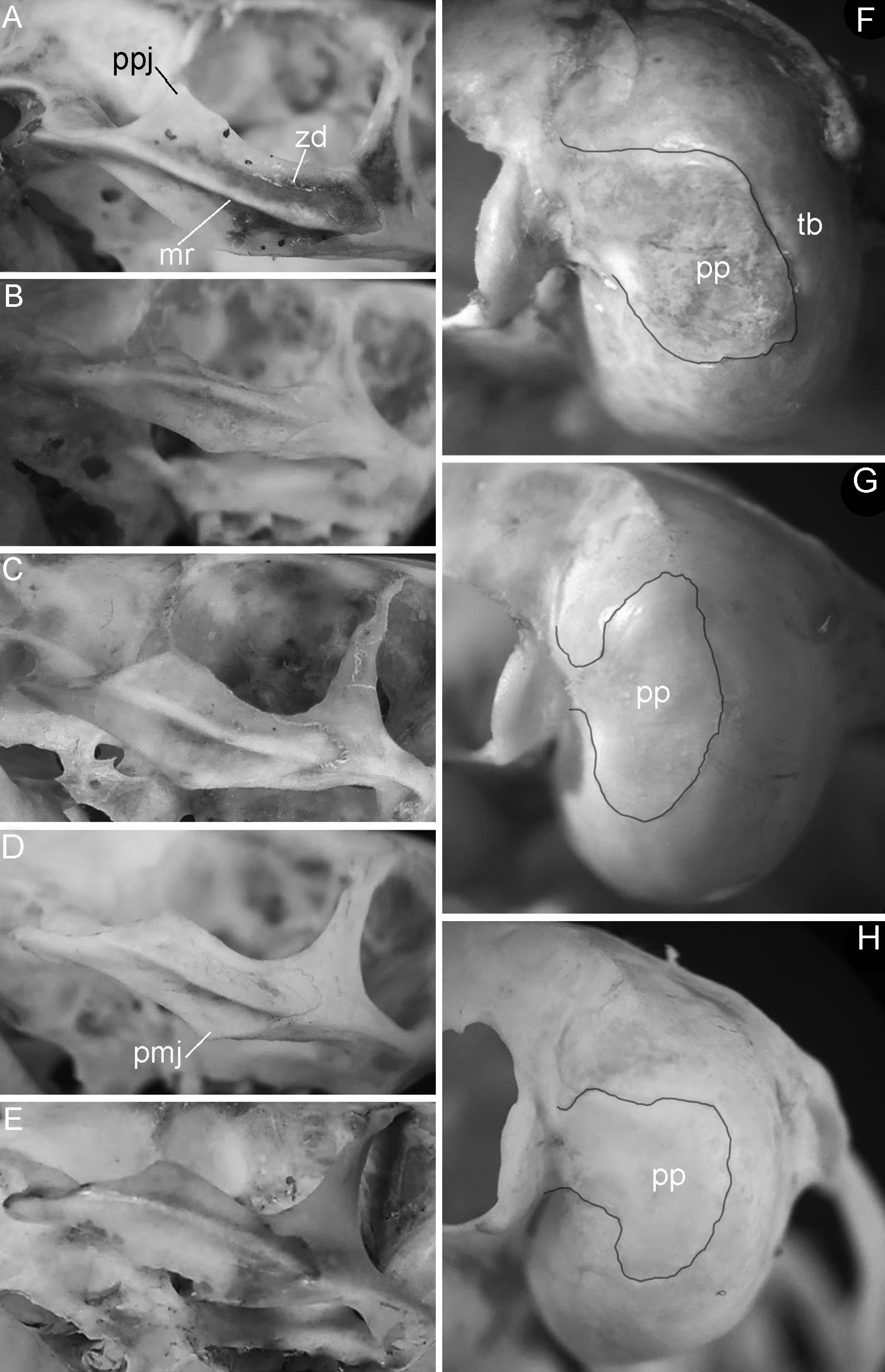


**Fig. 4S.** Selected cranial traits of *Ctenomys* (A, F, holotype of *C*. *bidaui* n. sp. [CFA 11867]; B, G, holotype of *C*. *c.* *contrerasi* n. subsp. [CFA 11853]; C, holotype of *C*. *contrerasi* *navonae* n. subsp. [CNP 1043]; D, H, holotype of *C*. *thalesi* n. sp. [CFA 11849]; and E, holotype of *C. sericeus* [USNM 84189]): A-E) zygomatic arches in lateral view; G-I tympanic bullae (tb) in posterolateral view. Abbreviations: mr, masseteric ridge; pmj, mandibular process of jugal; pp, paraoccipital process; ppj, postorbital process of jugal; zd, zygomatic depression. Figures are not in scale to facilitate comparisons.


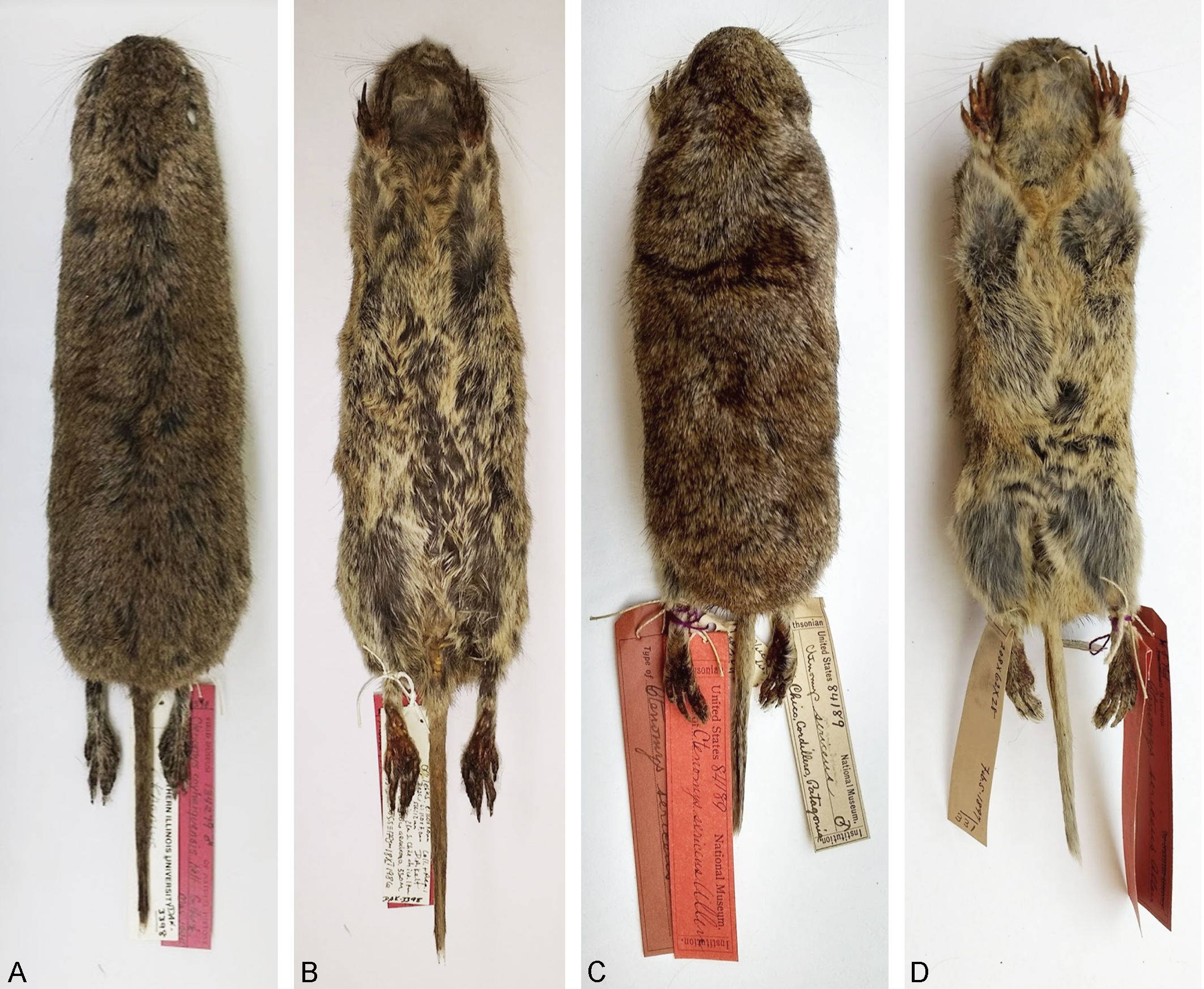


**Figure 5S.** Dorsal (A, C) and ventral (B, D) views of the skins of the holotypes of *C. coyhaiquensis* (A, B; FMNH 134279), and *C. sericeus* (C, D; USNM 84189). Figures are not in scale.

**
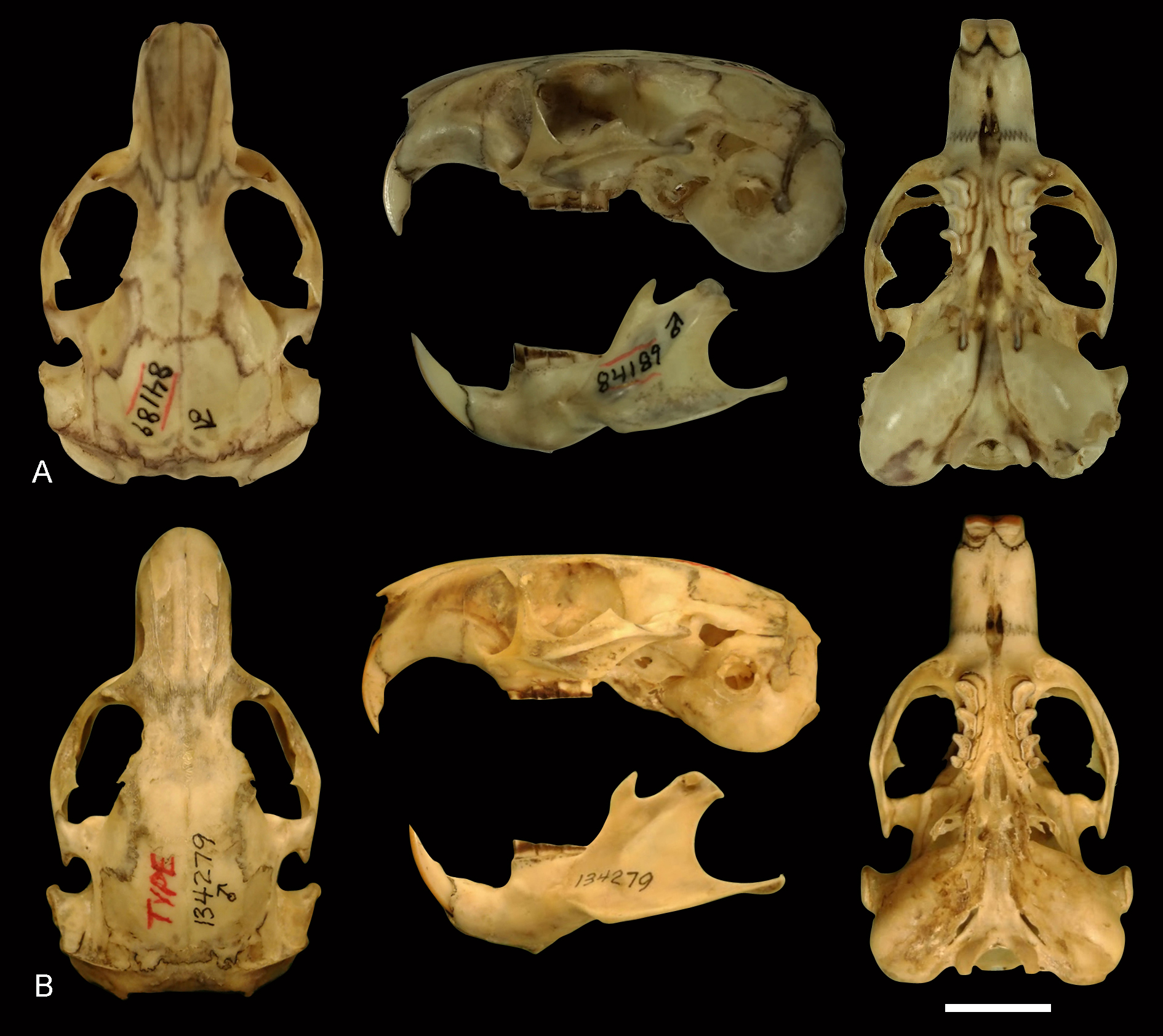
**

**Figure 6S.** Dorsal, lateral and ventral views of skulls and labial views of the mandibles of the holotypes of *Ctenomys sericeus* (A; USNM 84189) and *C. coyhaiquensis* (B; FMNH 134279). Scale = 10 mm.
